# Peptide Property Prediction for Mass Spectrometry Using AI: An Introduction to State of the Art Models

**DOI:** 10.1002/pmic.202400398

**Published:** 2025-04-10

**Authors:** Jesse Angelis, Eva Ayla Schröder, Zixuan Xiao, Wassim Gabriel, Mathias Wilhelm

**Affiliations:** ^1^ Computational Mass Spectrometry Technical University of Munich Freising Germany; ^2^ Munich Data Science Institute (MDSI) Technical University of Munich Garching Germany

**Keywords:** deep learning, machine learning, mass spectrometry, peptide property prediction, proteomics

## Abstract

This review explores state of the art machine learning and deep learning models for peptide property prediction in mass spectrometry‐based proteomics, including, but not limited to, models for predicting digestibility, retention time, charge state distribution, collisional cross section, fragmentation ion intensities, and detectability. The combination of these models enables not only the in silico generation of spectral libraries but also finds many additional use cases in the design of targeted assays or data‐driven rescoring. This review serves as both an introduction for newcomers and an update for experienced researchers aiming to develop accessible and reproducible models for peptide property predictions. Key limitations of the current models, including difficulties in handling diverse post‐translational modifications and instrument variability, highlight the need for large‐scale, harmonized datasets, and standardized evaluation metrics for benchmarking.

**Abbreviations**
AA: amino acid
AUC: area under the curve
BiGRU: bidirectional gated recurrent unit
BiLSTM: bidirectional long short‐term memory
CCS: collisional cross sections
CID: collision‐induced dissociation
CNN: convolutional neural network
CS: charge state
CSD: charge state distribution
DDA: data‐dependent acquisition
DIA: data‐independent acquisition
DT: decision tree
ETD: electron‐transfer dissociation
FDR: false discovery rate
GRU: gated recurrent unit
IMS: ion mobility spectrometry
iRT: indexed retention time
LSTM: long short‐term memory
*m*/*z*
: mass‐to‐charge ratios
MAE: mean absolute error
MAPE: median absolute percent error
MLP: multi‐layer perceptron
MS: mass spectrometry
MSE: mean squared error
OOD: out‐of‐distribution
PCC: Pearson correlation coefficient
PTM: post‐translational modification
RMSE: root mean squared error
RNN: recurrent neural network
RT: retention time
SVM: support vector machines

## 1 Introduction

In recent years, the interest in artificial intelligence (AI) has skyrocketed. With prominent examples such as *ChatGPT* [1], *StableDiffusion* [2], and *Gemini* [3], the buzzword AI can be found in many popular media headlines [4, 5]. This cultural phenomenon continues within the scientific domain, where AI is revolutionizing research across different fields. From accelerating drug discovery [6, 7] to improving climate modeling [8, 9], its potential to boost scientific discoveries is becoming increasingly clear. This is underscored by the recent Nobel Prize awarded for the development of AI in biochemistry [10]. For this progress to continue, however, it is critical that researchers stay informed about the latest developments in their field. It is also important that the field remains accessible to a wide range of talent. Inclusivity fosters innovation and encourages the diverse perspectives essential for groundbreaking discoveries that benefit society.

One area profoundly impacted by advances in AI is bioinformatics, particularly in the field of proteomics, where AI models analyze massive data sets and identify patterns and correlations that were previously undetectable [11]. The benefits of AI for proteomics are especially significant in the prediction of peptide properties. Here, it can be used to predict the behavior of peptides in mass spectrometry (MS) experiments. By predicting peptide properties, it is possible to generate expected in silico spectra of known and unknown peptides, allowing for more accurate identification and quantification [12]. To this end, the most commonly studied properties in the field are retention time and fragment ion intensities, while other properties such as precursor charge state distribution (CSD) or protein digestibility are less frequently studied.

### 1.1 Aim

In this review, we will follow the workflow of a proteomic MS experiment to demonstrate how AI is applied in current prediction models. While the models we focus on have either self‐reported performance gains over recent counterparts or have been identified as superior in comparative studies, it is important to note that this does not mean they are universally optimal. Other models may perform better in certain scenarios or under different conditions. Our goal is twofold: First, to guide new talent into this exciting field, and second, to update more experienced researchers about recent advances beyond the key property (or properties) on which they may currently be focused on. To achieve the first goal, we aim to make the field more accessible by briefly explaining how the key layers of AI models work in the context of selected examples across different peptide properties. This approach ensures that newcomers can develop an intuitive understanding of the fundamental components and how they are applied in real‐world scenarios. The selection of models attempts to cover a wide range of different approaches, architectures, and methodologies to show the diversity of techniques used in peptide property prediction. To keep the barrier of entry as low as possible, we included background information on the fundamentals of machine learning (Supporting Information). While not aiming to be exhaustive to achieve the second goal, we provide a comprehensive collection of the models developed in recent years to provide a clear picture of the field's current state (Table S1). In doing so, we not only highlight the strengths and applications of these tools but also discuss their limitations and identify promising directions for future research.

This paper focuses on models for predicting peptide properties in the context of standard MS‐based proteomics experiments or properties that influence MS‐based proteomics, building on prior work [11, 12, 13, 14], which we encourage to read as well. For work focusing on biomedical properties, such as classification of MHC‐binding peptides, we refer to other reviews [15, 16] with this specific focus. We also do not focus on specialized MS‐based proteomics approaches like for cross‐linked peptides (e.g., *pDeepXL* [17]) or glycopeptides (e.g., *DeepGlyco* [18]). Although our focus is on MS, many of the techniques discussed here are also applicable to other domains, such as biomedical properties.

### 1.2 Limitations in Model Evaluation and Comparison

The process of building and evaluating machine learning (ML) models involves five critical components: (1) training data, including preprocessing steps; (2) model architecture; (3) loss function; (4) evaluation data; and (5) evaluation metrics. Changes in any of these components can significantly influence a model's apparent performance, making fair comparisons between models inherently challenging. This difficulty is exacerbated by the lack of universally accepted benchmark datasets or standardized training sets for the peptide properties discussed in this review. While datasets such as *ProteomeTools*/*PROSPECT* [19], which provide synthetic data with reliable labels, and the nine‐species dataset [20] are available, none of these have been consistently adopted across studies. As a result, it becomes nearly impossible to determine which models are truly “better” or “best” and what specific changes contributed to improved performance. For example, consider two models evaluated using the mean absolute error (MAE) metric. One model is trained using an MAE loss function, while the other uses a mean squared error (MSE) loss function on the same dataset. The first model is likely to perform better on the MAE metric, because it was optimized for that specific criterion.

Furthermore, similarity between training and evaluation datasets can significantly influence model evaluation outcomes. Models trained on data that is similar to the evaluation dataset may appear to perform better, not because of superior architecture or methodology, but due to overfitting to specific characteristics of the data. Given these complexities, this review refrains from making judgments about which models, architectures, or approaches are objectively “better” or “best.” Instead, it highlights the challenges of evaluating and comparing models within this domain.

## 2 MS‐Based Peptide Property Prediction

A standard MS‐based proteomics experiment involves several steps: protein digestion, chromatographic separation, peptide ionization, sometimes followed by optional gas‐phase separation, and peptide fragmentation. This approach is commonly referred to as bottom‐up or shotgun proteomics [21]. Accurate prediction of peptide behavior at various stages of this process can increase confidence in peptide identification and help detect peptides that would otherwise be missed, with both aspects having implications for protein quantification [22]. In data‐independent acquisition (DIA) workflows, the shift toward predictive models has become essential in recent years, moving beyond reliance on experimental libraries alone [23]. Empirical spectral libraries provide key information from experiments, such as the proteins and peptides that may be present, charge states (CS), retention times (RT), and fragmentation patterns, but they are limited, especially in their ability to represent all potential variants. While data‐dependent acquisition (DDA) has traditionally not relied on libraries due to their incomplete nature, targeted approaches, and DIA have become more reliant on them. This reliance has likely led DIA to adopt predictive modeling techniques more quickly. However, DDA is also increasingly shifting towards these methods, driven by advances in computational tools and the need for more comprehensive peptide identification and quantification.

### 2.1 Digestibility

#### 2.1.1 Background

After protein extraction, which we will not cover in this review, the first step in MS‐based proteomics typically involves the enzymatic digestion of proteins. Specific proteases cleave the proteins at designated sites into smaller peptides. These peptides are then analyzed to identify and quantify their parent proteins [24, 25]. The process of enzymatic digestion is stochastic, meaning that each potential cleavage site in a protein has a specific probability of being targeted by the protease. Rather than cutting equally at every possible site, proteases preferentially cleave certain sites, resulting in different peptides being produced in each digestion event [26]. In theory, given infinite time, the protease would eventually cleave all possible sites, resulting in a consistent outcome. In practice, however, external factors like time, temperature, and pressure as well as peptide properties like their 3D structure impact the digestion process [25, 27, 28]. This variability influences the final set of peptides available for analysis, affecting both the efficiency and coverage of protein identification in proteomics experiments, a principle exploited by limited proteolysis‐coupled mass spectrometry (LiP‐MS) [26]. Considering the likelihood of peptide generation during enzymatic digestion helps to predict which peptides will appear in the analysis [25, 27] and, thus, may provide additional evidence for the presence of certain proteins in a sample [28].

**FIGURE 1:**
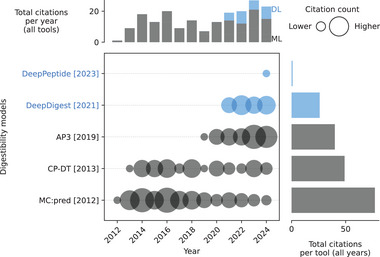
Citations over time for different digestibility models. As of February 18, 2025. The cutoff years are 2010 and 2024. References to all models can be found in Table S1. Models using deep learning (DL) are blue. Models using only classical machine learning (ML) are gray. The exact values of the citation counts per tool and year can be found in Table S2. Created with https://github.com/jesseangelis/Citation_vis/ and *OpenAlex* [32].

Different models have been proposed to predict the digestibility of peptides (Table S1). The earlier ones are based on classical machine learning methods like support vector machines (SVM) in *MC:pred* [29] or decision tree (DT)‐based methods like *CP‐DT* [30] or *AP3* [27]. Recently, however, there has been a shift toward DL models applying more complex architectures (Figure 1) [25, 31].

#### 2.1.2 Introduction to *DeepDigest*

**FIGURE 2:**
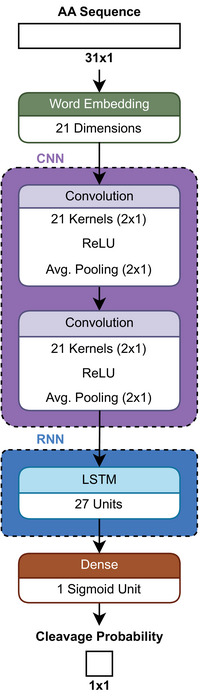
Schematic architecture of *DeepDigest* [25].

One of these modern deep learning approaches, called *DeepDigest* [25], predicts cleavage sites in protein sequences and their respective probabilities based on the chosen protease. The architecture of *DeepDigest* (Figure 2) is designed to consider that proteolytic digestion is influenced not just by the sequence at the cleavage site itself, but also by the surrounding amino acids. During its development, it was found that including more neighboring amino acids improved the model's performance, but this improvement plateaued at about 15 amino acids on either side of the cleavage site. To utilize this information, sequences of 31 amino acids are embedded in a 31 × 21 matrix using word embedding (see Supporting Information). The embedding is fed into a convolutional neural network (CNN) with average pooling to capture the relationships between amino acids and to detect discriminative patterns [25].

CNNs are extensively used in computer vision, where they have significantly improved the ability to identify patterns and features that represent different objects in images [33, 34]. Following the CNN, a type of network known as long short‐term memory (LSTM) is used to capture the dependencies between amino acids based on their positions in the sequence [25]. LSTMs are designed to retain information over long sequences, allowing the model to consider how the presence of one amino acid affects the characteristics of the next one in the sequence. They are a type of recurrent neural network (RNN) and, in principle, able to handle inputs of variable lengths [35]. However, in practical settings, the input is often padded to have a constant length [25]. The outputs of the LSTM are combined in a dense layer (also called a fully connected layer) to form a single value. Finally, a sigmoid function is applied to this output, resulting in a value between 0 and 1 for each 31‐mer, indicating the probability of a cleavage event occurring at that specific site [25].

During the development of *DeepDigest*, the challenge of unbalanced data became apparent, where the number of used cleavage sites significantly outweighed the number of missed (uncleaved) cleavage sites. Such an imbalance significantly impacts the training process by affecting the loss (see Supporting Information). Specifically, the model is not penalized as often or as strongly for incorrect predictions on the underrepresented class because it encounters these instances less frequently during training. As a result, the model fails to learn how to effectively predict the minority class, leading to poorer performance on that class. This problem is not specific to digestibility and is likely a source of error in many peptide property prediction models. To address this issue, a balanced cross‐entropy loss function has been implemented for *DeepDigest*:

(1) $$L\left(y,p\right)=-\alpha \cdot y\cdot \log\left(p\right)-\left(1-\alpha \right)\cdot \left(1-y\right)\cdot \log\left(1-p\right)$$

where y ∈ {0, 1} is the ground‐truth of the cleavage site, p ∈ [0, 1] is the predicted cleavage probability, and α ∈ [0, 1] is an “empirically” adjusted class weight for y [25]. Although it was not explained what “empirical” means, it likely refers to the inverse frequency of the classes, making the less frequent class more important. This approach improved the model's sensitivity to the less frequent cleavage sites, leading to a more robust and reliable prediction framework.

For the base model of *DeepDigest*, the area under the curve (AUC) values (see Supporting Information) ranged from 0.849 to 0.978 across different proteases. The model was further improved through transfer learning (see Supporting Information), where it was first pre‐trained on a large dataset and then fine‐tuned on smaller, protease‐specific datasets to account for varying experimental conditions. This approach led to a significant increase in performance, improving AUC values by up to 13.6% for the lowest‐performing protease, LysN. To validate the model, it was compared to *MC:pred* on four different trypsin datasets. *DeepDigest* consistently outperformed *MC:pred*, with AUC scores of 0.967, 0.987, 0.987, and 0.985, compared to 0.961, 0.945, 0.972, and 0.935 for *MC:pred* [25].

#### 2.1.3 Limitations and Future Directions

The *DeepDigest* publication implies that the input sequences are restricted to unmodified amino acids, while the training data include fixed and variable modifications depending on the dataset [25]. This inconsistency could introduce bias, as the model's predictions depend not only on the sequence itself but also on the experimental conditions represented in the training data. However, we were unable to verify that the input is truly restricted to unmodified amino acids, as the model is only available for download over an insecure HTTP connection. Since post‐translational modifications (PTMs) can inhibit protease activity and thus affect proteolysis [36], it is essential to support input of modified amino acid sequences. This adaptation would allow the model to predict digestion more accurately for any given sequence, rather than being confined to the conditions of the training data.

Another potential bias source is that peptide quantification is limited to those peptides detectable in mass spectrometry analysis. If a peptide is unlikely to be detected, due to poor ionization or other factors, it will be marked as absent, even if it is present in the digested sample [25]. This discrepancy could distort the labeling, making it no longer a reliable ground truth. We will only briefly mention this issue here and revisit it in detail in the section on detectability.

*DeepDigest* provides a modern approach to digestibility prediction and performs well compared to previous models. However, there are a few areas for potential improvement. First, its accessibility could be enhanced by adopting common practices, such as making it available on a trusted platform like *GitHub*. Following the FAIR (findable, accessible, interoperable, reusable) principles is important for any machine learning model [37]. Second, allowing the specification of PTMs in the input would help reduce biases introduced by experimental conditions. Finally, extending digestibility predictions to include peptides beyond those detectable by mass spectrometry would offer a more complete view. Addressing these areas could make future models even more reliable and comprehensive.

### 2.2 Retention Time

#### 2.2.1 Background

After proteins are digested into individual peptides, they are separated using liquid chromatography, primarily to reduce the sample complexity. There are several types of liquid chromatography, such as reverse‐phase [38], ion‐exchange [39], and size‐exclusion [40], each suited to specific peptide characteristics. The time it takes for each peptide to pass through the chromatography system, known as the retention time (RT), depends on how the peptide interacts with the stationary phase of the column, the mobile phase, other analytes, and other system parameters (e.g., temperature and pressure). These interactions are influenced by factors such as the peptide hydrophobicity, size, conformation, and the experimental conditions [41, 42]. In most cases, bottom‐up proteomics experiments use a reversed‐phase column in‐line with a mass spectrometer [41].

Knowledge of a peptide's retention time is essential, as it significantly increases confidence in its identification [43]. It is also crucial for targeted DIA analyses, as it allows for the scheduling of peptide precursor extraction windows, improving both specificity and throughput [44]. Databases containing known retention times for each peptide, ideally matching the experimental setup, are therefore needed. However, creating such databases is time‐consuming and expensive due to the large variety of peptides and LC conditions [45].

To overcome the limitation of varying retention times between different runs, the indexed retention time (iRT) was introduced [46]. This metric indicates the retention time of a given peptide relative to a set of reference iRT‐peptides, allowing for the alignment of different measurements and more accurate predictions [46]. Most RT predictors today use some form of normalized RT, most commonly iRT, to harmonize the training data and to achieve some degree of generalization [47, 48]. Consequently, predicted iRTs often require calibration with experimental data to provide meaningful real‐world insights [49].

**FIGURE 3:**
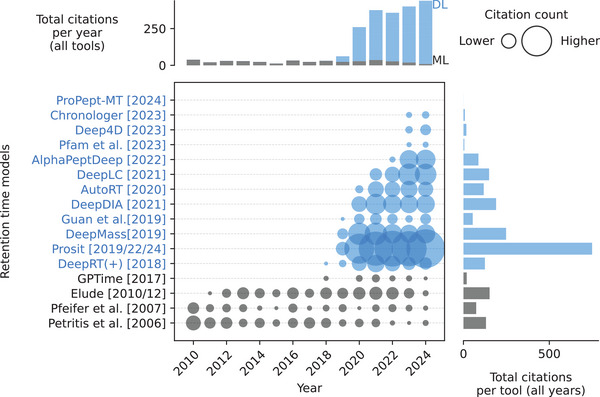
Citations over time for different retention time models. As of February 18, 2025. The cutoff years are 2010 and 2024. *Chronologer* is a preprint. *Prosit* includes publications from 2019, 2022, and 2024 (preprint). *Elude* includes publications from 2010 and 2012. References to all models can be found in Table S1. Models using deep learning (DL) are blue. Models using only classical machine learning (ML) are gray. The exact values of the citation counts per tool and year can be found in Table S2. Created with https://github.com/jesseangelis/Citation_vis/ and *OpenAlex* [32].

Peptide RT prediction models have evolved significantly, transitioning from early machine learning approaches like multi‐layer perceptrons (MLPs) and SVMs to more sophisticated deep learning techniques (Figure 3 and Table S1). The shift began with *DeepRT(+)* [50], which uses CNNs, followed by *Prosit* [51], which uses an architecture known as bidirectional gated recurrent units (BiGRU) in combination with the so‐called attention mechanisms. This DL trend continued with models such as *DeepLC* [48], using CNNs, and *AlphaPeptDeep* [47], which incorporates BiLSTMs and attention. While model complexity has increased, the input focus remains on peptide sequences, with variations in loss functions such as MSE, MAE, and root mean squared error (RMSE) (see Supporting Information). A notable trend is the rise of open‐source development, with many models now available on *GitHub*. Recent models, including *Prosit*, *AlphaPeptDeep*, and *DeepLC*, are rapidly gaining traction, with *Prosit* seeing a sharp increase in citations, from 20 in 2019 to 181 in 2024. However, it is important to note that *AlphaPeptDeep* and *Prosit* do not exclusively predict retention time, which leads to higher citation counts, as they have broader applicability.

#### 2.2.2 Introduction to *DeepLC*

**FIGURE 4:**
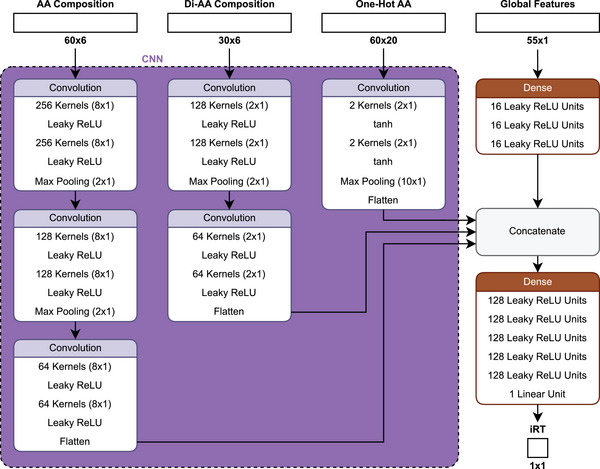
Schematic architecture of *DeepLC* [48].

*DeepLC* [48] uses an architecture (Figure 4) that aims to handle PTMs that are not part of the training set. This is approached by three additional inputs to a one‐hot encoding (see Supporting Information) of amino acids as follows: first, an amino acid composition matrix encoding the number of atoms (C, H, N, O, P, and S) of each amino acid. Second, a diamino acid composition matrix that does the same, but for two amino acids at a time. These two‐atom count embeddings allow for the representation of modified amino acids by adding the respective atom counts of the modification. Third, a global numerical feature list, including, for example, the length and mass of the peptide. Each input takes its own path within the network, where all but the global feature inputs are subjected to convolutional layers. The paths are then combined and processed by multiple dense layers. The model is optimized using the MAE as a loss function [48].

The multi‐input approach was successful in predicting most modifications that were not included in the training set, but it was not successful for some, particularly phosphorylation and nitrotyrosine modifications. This could be due to the different chemical structures of these modifications compared to those in the training set [48]. It suggests that the model may be overfitting or experiencing data leakage from the modifications used during training, limiting its ability to predict modifications that are chemically distinct. This limitation may arise from an inadequate feature representation or insufficient coverage of the relevant chemical space in the training data, hindering effective generalization. Ultimately, it highlights an ongoing challenge with out‐of‐distribution (OOD) problems for PTMs, despite efforts to address it.

#### 2.2.3 Introduction to *Chronologer*

As mentioned above, deep learning methods require substantial amounts of training data and typically assume that these data are accurate. However, proteomics data are often imperfect and likely to contain incorrect labels, as it is commonly derived from database search results filtered to a 1% false discovery rate (FDR). Furthermore, creating large datasets, covering the physicochemical space of interest as best as possible, can be cumbersome and expensive [52]. In a recent preprint model called *Chronologer* [49], these issues were addressed by creating a harmonized dataset from multiple experiments and training a model that considers the error in the data. The datasets included those used to train other models such as *DeepLC* and *Prosit*. Harmonization was not done by linearly aligning the gradient using shared reference peptides across all datasets, but by separately predicting the iRT for all *Prosit*‐predictable peptides in each dataset. A monotonic function was then fitted to align the measured retention times to the respective predicted retention times from *Prosit* (the *Prosit* prediction space). Using this function, it was possible to transform all measurements into the *Prosit* prediction space and thus harmonize all datasets to the same global reference without the need for a shared standard across datasets. The harmonized data were then aligned to a physiochemically meaningful dimension, representing the hydrophobic index of a reversed‐phase high‐performance liquid chromatography. This created a database containing ∼2.2 million unique peptides measured under different experimental setups. In addition, the generated dataset contained 10 different PTMs [49].

*Chronologer*’s residual CNN was trained using a loss function that dynamically masks datapoints that fall outside of the 99% confidence interval when calculating the errors. To select a good base loss function, harmonized measurements of the same peptides were compared across different experimental conditions. These differences were then analyzed to see whether they followed a Gaussian distribution (related to MSE) or a Laplacian distribution (related to MAE). The analysis found that the empirical distribution was better described by a Laplacian distribution, so the MAE was chosen as the loss function. Together with the masking approach, this resulted in a significant improvement in performance, achieving an MAE of 0.81 compared to the reported MAEs of 1.29 for *DeepLC*, 1.27 for *Prosit*, and 1.48 for *AlphaPeptDeep* [49].

#### 2.2.4 Limitations and Future Directions

While *Chronologer* is not able to predict unseen modifications out of the box, it has been shown that new modifications can be learned relatively quickly using transfer learning, without requiring excessive training data [49]. *DeepLC* has demonstrated the ability to predict retention times for peptides with modifications that are chemically similar to those in its training set [48]. However, to our knowledge, no study has shown convincing results indicating that any model can perform well on peptides with chemically dissimilar modifications. Bearing in mind that the *Chronologer* results have not yet been peer‐reviewed, they show how an error function adapted to the analyzed data can improve the performance of a model. This data‐aware approach could be valuable for other properties as well.

### 2.3 Charge State Distribution

#### 2.3.1 Background

After chromatographic separation, the sample enters the ion source where peptides are ionized, most commonly by electrospray ionization. The number of charges carried by a peptide is called its charge state (CS). Peptides can exhibit various CS depending on, for example, their amino acid composition and order, their length, and their modifications [53, 54, 55, 56]. The CS is also influenced by experimental factors like the ionization method, the LC mobile phase, and the complexity of the sample matrix [34, 57–61]. But even peptides with the same sequence can have different CS within a single experiment. The ratio of different CS for the same peptide sequence is called charge state distribution (CSD) [53, 62, 63]. Since mass spectrometers measure ions based on their mass‐to‐charge ratios (*m*/*z*), predicting the dominant CS and CSD is critical for building accurate spectral libraries, aiding in peptide identification during MS analysis [53].

**FIGURE 5:**
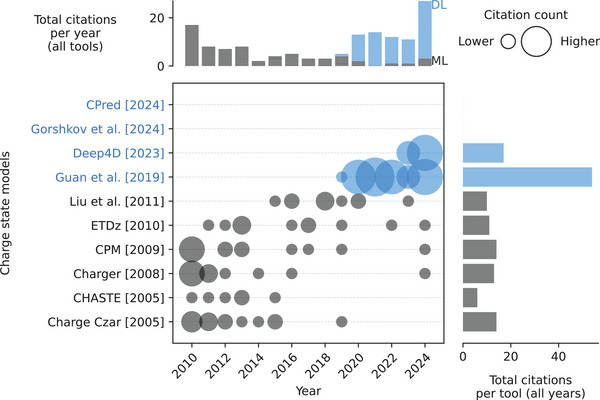
Citations over time for different charge state and charge state distribution models. As of February 18, 2025. The cutoff years are 2010 and 2024. *AlphaPeptDeep* is not included as its predictor has not been part of a publication yet. *CPM* is an abbreviation for *Charge Prediction Machine*. References to all models can be found in Table S1. Models using deep learning (DL) are blue. Models using only classical machine learning (ML) are gray. The exact values of the citation counts per tool and year can be found in Table S2. Created with: https://github.com/jesseangelis/Citation_vis/ and *OpenAlex* [32].

Compared to other properties, such as retention time, there has only been limited published work aimed at predicting CS or CSD of peptides using machine learning (Figure 5 and Table S1). By 2010, several basic machine learning models utilizing linear regression, or SVM, had been developed in the context of electron‐transfer dissociation (ETD) fragmentation. Here either the highest possible or the most abundant precursor charge state given a particular ETD spectrum is estimated. Thus, these models use MS2 spectra as input, often manually curated, which limits the complexity of the training data [64, 65, 66, 67, 68, 69]. In 2019, the Guan et al. model started the deep learning era for more general precursor CS prediction by being the first one on several ends as follows: (1) changing the input to peptide sequences which included some PTMs. (2) Using MS1‐based extracted ion chromatogram data to cover as much of the underlying CSD as possible. (3) Using a BiLSTM model to predict precursor CSD [62]. This development led to an increased interest in CSD prediction.

Building on Guan et al. and retaining most of the concepts, the recent models Gorshkov et al. [53], and *CPred* [63] reported improvements of various aspects. The former focuses on the MS1‐based data processing and the loss function, and the latter focuses on PTM integration and feature engineering [53, 63]. In parallel to these developments, several other deep learning‐based CS prediction models have been developed to support CCS or MS2 spectra prediction and in some cases are mentioned as sidenotes in the respective main publications. They differ from the above CSD prediction models by either predicting only a single CS or a binary representation of a limited number of CS and are copies of the architecture used for the main prediction task of the publication [70, 71, 72].

#### 2.3.2 Introduction to *CPred*

**FIGURE 6:**
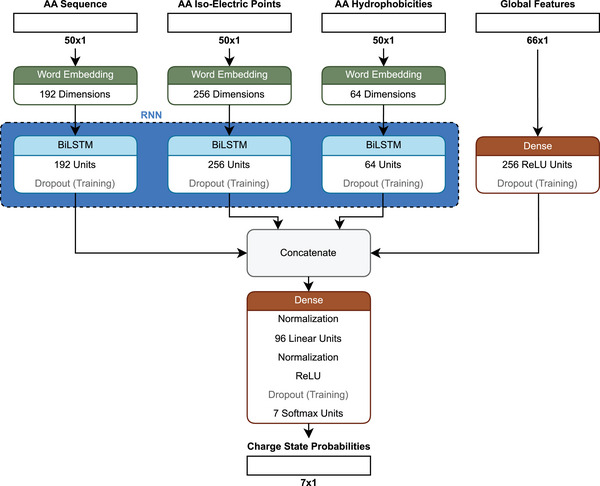
Schematic architecture of *CPred* [63].

*CPred* is a model that uses a bidirectional long short‐term memory (BiLSTM) architecture (Figure 6) [63]. A BiLSTM is similar to the already mentioned LSTM in its ability to capture contextual relationships between amino acids in a sequence. However, while an LSTM processes the sequence in a single direction (from start to end), a BiLSTM reads the sequence in both directions, thus extracting information from the beginning and the end simultaneously. This bidirectional approach enables the model to gain a more comprehensive understanding of the sequence by considering both the left and right context [73]. To encode the peptides, a similar approach to the one described for *DeepLC* was followed where amino acids are reportedly one‐hot‐encoded in addition to providing the atomic compositions separately to capture PTMs. Contrary to *DeepLC*, *CPred* does not include atom counts per amino acid, only calculating the peptide‐wide atom count. Other static features describing peptide‐wide characteristics are, for example, peptide length and the fraction of basic amino acids. The sequential features are the already mentioned one‐hot‐encoded amino acids and the per‐amino acid specific characteristics: hydrophobicity, and isoelectric point. The loss function is the MSE [63].

In contrast to the Guan et al. model, *CPred* does not use MS1‐based, but MS2‐based CSDs. A strong class imbalance in the data was noted, observing that the training data had higher probabilities for +2 and +3 charges compared to, for example, +5 or +7. As a result, the model is not able to accurately predict the most abundant CS, if it is expected to be higher than +4. In the study, it was suggested to retrain the model with a balanced data set [63]. However, it might also be possible to adjust the loss function to address the class imbalances. An example could be the weighted cross entropy loss, as mentioned in the digestibility section. Here the entropy (the difference between a known and a predicted probability) could be weighted by the inverse average probability of each CS [74].

*CPred* was evaluated based on the median Pearson correlation coefficient (PCC) (see supporting information) between the predicted and experimental probabilities across all charge states. A value of 0.9997 was reported [63]. This metric, however, might not be suitable for the highly imbalanced dataset as it does not represent the infrequent classes of CS larger than +4 well. A CS wise evaluation might be more suitable or if a single value is preferred, the mean of the CS wise correlations.

PTMs were found to mostly have minor impacts on the performance, however, the results in the *CPred* study indicate that some modifications, such as trimethylation, GlyGlycylation, crotonylation, and biotinylation, show lower PCCs. An in‐depth analysis on this issue was not provided [63], but these lower PCCs raise concerns about the generalizability of the model to unseen modifications, as these specific PTMs differ more structurally from other lysine modifications. For instance, crotonylation is the only lysine‐PTM in the *CPred* set with an alkene bond, trimethylation is unique in possessing a quaternary ammonium group (although dimethylations can also have a quaternary ammonium, it is less common), GlyGlycylation is distinguished by a peptide bond, and biotinylation contains the distinct biotin structure. These structural differences suggest that *CPred* might struggle to generalize to unknown chemical features of PTMs, particularly when most lysine modifications in the dataset have different features. While one might argue that the well performing TMT6‐plex should also fall into this category, it stands apart due to its significant representation in the dataset, with 192,452 observations. This is far more than the other PTMs, most of which have fewer than 500 observations. For a study that investigates the impact of different PTMs on retention time, CS, and fragmentation we refer to [75].

A major point that needs to be considered when discussing any deep learning model, here highlighted on *CPred*, is that we observed sometimes significant discrepancies between the information provided in a manuscript and the implemented code. As mentioned above for *CPred*, the sequential inputs were reported to be the one‐hot encoded amino acid sequence, as well as the per amino acid isoelectric points and hydrophobicities. However, the supplied code shows that the amino acid sequence is not one‐hot encoded but encoded using word embedding. Further, while the per amino acid isoelectric points and hydrophobicities are used as input they are fed into a word embedding layer, effectively turning the float values into integers and using these integers as indices for the learned embeddings, thus limiting the information input to the rounded down values. This is especially significant for hydrophobicity as these values range between −0.7 and 1. These discrepancies highlight the need for a standardized reporting and common standards for supplied code, allowing reviewers to easily find and highlight such instances. Until this is part of the publication process, checking the source code of models before relying on them is advisable.

#### 2.3.3 Limitations and Future Direction

Due to the median PCC, used by many studies in this area, being an unsuitable metric for determining how well the present models compare against each other, further studies are needed to assess this issue. Generally, the different models for CS and CSD prediction are not directly comparable due to the many variations ranging from training data via label preparation to architectures. Like with *DeepLC*, the *CPred* study results suggest that simply adding atom counts does not enable accurate predictions for all unknown peptide modifications. This approach falls short when the modifications have chemically distinctive features from those in the training data. Further, like with *DeepDigest*, new standards required for publication should be implemented in order to ensure a high quality of models that allow for further development.

### 2.4 Ion Mobility

#### 2.4.1 Background

LC‐based separation is the preferred method for separating peptides to decrease sample complexity. Yet, some peptides might not be separable by the standard setup used for online LC. Ion mobility spectrometry (IMS) can in some cases separate these peptides, even if they are isomers, based on their different conformations and collisional cross sections (CCS), enabling more refined analysis of complex mixtures [76]. IMS instruments are classified into temporal dispersive, spatial dispersive, and confinement/selective release types, with techniques like drift tube ion mobility spectrometry (DTIMS), traveling wave ion mobility spectrometry (TWIMS), differential mobility spectrometry (DMS), field asymmetric ion mobility spectrometry (FAIMS), and trapped ion mobility spectrometry (TIMS) [77]. TIMS, a common version of IMS used today, works by guiding ions along an electric field with an inert buffer gas flowing into the opposite direction. This gas, usually helium or nitrogen, facilitates the different drift speeds of ions by colliding with them [78].

**FIGURE 7:**
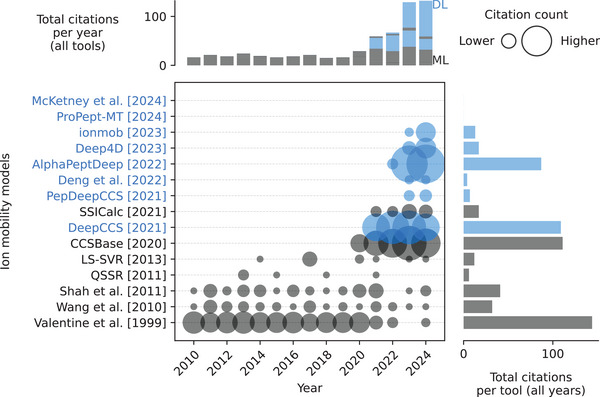
Citations over time for different ion mobility models. As of February 18, 2025. The cutoff years are 2010 and 2024. McKetney et al. is a preprint. *DeepCCS* is an abbreviation for *DeepCollisionalCrossSection*. References to all models can be found in Table S1. Models using deep learning (DL) are blue. Models using only classical machine learning (ML) are gray. The exact values of the citation counts per tool and year can be found in Table S2. Created with: https://github.com/jesseangelis/Citation_vis/ and *OpenAlex* [32].

The research landscape of peptide ion mobility prediction indicates an upward trend in research activities and citations over time (Figure 7). Some of the first attempts predicted the drift time, an indication of ion mobility [79]. Initially, a series of traditional ML methods such as single hidden layer neural network [80], partial least square [81, 82], support vector regression [82, 83, 84], and Gaussian process [82] combined with extensive feature engineering on the physico‐chemical properties were published. In recent publications from 2021 to 2024, a notable shift has occurred, transitioning from traditional methods to more advanced deep learning architectures, including CNNs, BiLSTMs, and Transformers, which aim to predict the peptides CCS (Figure 7 and Table S1) [47, 70, 71, 85–88]. Nowadays the common input types across models include sequence information and charge state. Regarding training data, earlier studies relied heavily on in‐house datasets, whereas more recent models frequently utilize the dataset from Meier et al. [70]. Open‐source practices have also improved, with many recent models being released under licenses like MIT and Apache‐2.0, and their code being made available on platforms like *GitHub*. This code often supports both inference and training, reflecting a trend towards greater accessibility and usability in the research community.

#### 2.4.2 Introduction to *ionmob*

**FIGURE 8:**
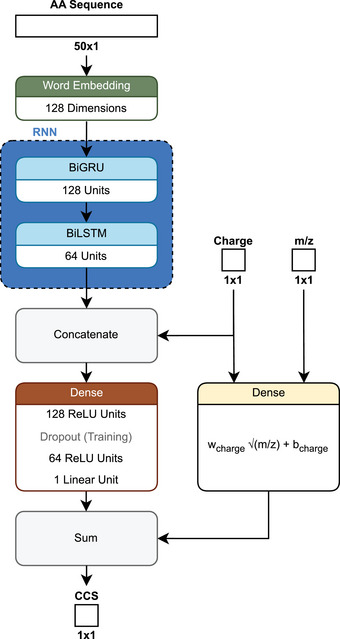
Schematic architecture of *ionmob* [86].

Since the CCS shows a strong correlation with the square root of *m*/*z* [78, 86], *ionmob* [86] approaches the task of predicting CCS by starting with exactly this square root of *m*/*z*. It is used as an initial projection, aiming to circumvent problems regarding unbalanced data, where higher charges are less frequent. It also allows comparison of the predictions against the baseline of the simple square root calculation and thus establishing a minimum required accuracy. The architecture of *ionmob* follows a dual‐path design (Figure 8). The main path takes the amino acid sequence and embeds it into a vector space using token embeddings (see Supporting Information). While the embedding process was not fully explained in the study, the text implies that individual amino acids were used as tokens, which would then be referred to as word embedding [86].

The embeddings are then processed by bidirectional gated recurrent units (BiGRU) [86]. Similar to LSTMs, GRUs are a type of recurrent neural network designed to capture long‐term dependencies in sequential data. The key difference between them lies in how they process and manage information. LSTMs use three gates, each governed by sigmoid functions, to control the flow of information. The input gate regulates how much new information is added to the memory, the forget gate determines how much of the previous information is discarded, and the output gate controls how much of the updated memory is passed to the next step. In contrast, GRUs simplify this process by using only two gates: the update gate, which balances the retention of previous information with newly computed information, and the reset gate, which controls how much of the previous information is used to compute the new state [89]. BiGRU, like BiLSTM, refers to a design where information flows in both directions. GRUs were shown to produce smaller errors compared to LSTMs in a study on traffic flow [89]. To our knowledge, however, it is unclear whether this holds true for peptide property predictions based on amino acid sequences.

In *ionmob* the output of the BiGRUs is processed by two dense layers which also incorporate information about the CS. This output is then combined with a learned square root projection of the mass to charge ratio as an initial projection to result in the predicted CCS value. Measured CCS were aligned to a reference dataset, and training was performed using the MAE between predicted and aligned measurements [86].

The performance was compared against three other recently published CCS predictors *AlphaPeptDeep* [47], *PepDeepCSS* [85], and *DeepCollisionalCrossSection* [70] based on the median absolute percent error (MAPE) (see Supporting Information) to account for differences in training data normalization. *ionmob* reported comparable performance with *PepDeepCSS* and *DeepCollisionalCrossSection* while *AlphaPeptDeep* performed slightly worse. Depending on the CS, the prediction error changes more strongly across all compared prediction models. Peptides observed with a charge state of +2, +3, and +4 exhibited MAPEs of around 1.1%, 1.9%, and 2.8%–8%, respectively [86]. This indicates that all models do not generalize well for charge states that appear less frequently in the training dataset. While the addition of an initial projection might have helped in addressing that problem, none of the models adjusted their loss functions for class imbalance [47, 70, 85, 86]. A balanced metric like a weighted MAE, where the weights correspond to the inverse frequency of each charge state might allow for predictions that generalize better.

It was not addressed how *ionmob* performs with previously unseen modifications, likely because it currently supports only a limited set of common modifications [86]. Based on *ionmob*’s source code, supported modifications include phosphorylation (serine, threonine, and tyrosine), N‐terminal acetylation (lysine), oxidation (methionine), carbamidomethylation (cysteine), and carbamylation (cysteine) [86].

#### 2.4.3 Limitations and Future Direction

As discussed in the previous properties, class imbalance seems to be a problem in current CCS prediction models too. The less frequent a given charge state is, the larger the error of the corresponding CCS gets. In general, this problem seems to be overlooked, as none of the above methods take measures to address it. Further, the problem of accurately predicting modified peptides remains unsolved for CCS. While *ionmob*, *PepDeepCCS*, and *DeepCollisionalCrossSection* only have a fixed limited number of modifications that are embeddable [70, 85, 86], *AlphaPeptDeep* employs a chemical composition approach similar to the ones discussed earlier [47]. However, the results in the *AlphaPeptDeep* study, regarding how well it performs on individual PTMs for CCS predictions, are not detailed enough to draw a conclusion about its performance. Therefore, further studies regarding the CCS predictions of peptides with unknown modifications are needed.

### 2.5 Fragmentation Ion Intensities

#### 2.5.1 Background

To increase sensitivity and specificity, tandem MS is used in most proteomic measurements. Here *m*/*z* bands containing selected peptide ions (precursor ions) are selected for fragmentation. This fragmentation can be performed in multiple ways [90, 91]; however, the commonly applied fragmentation in bottom‐up proteomics is collision‐induced dissociation (CID) in the form of resonance‐type CID (referred to as CID on Thermo Fisher instruments) or beam‐type CID (referred to as HCD on Thermo Fisher instruments or CID by most other vendors) [90, 91].

Predicting a peptide fragmentation spectrum can take one of two main approaches: (1) focusing on the prediction of backbone ions (i.e., a, b, c, x, y, and z ions, with or without neutral losses) or (2) predicting the complete spectrum, including non‐backbone ions (e.g., immonium ions, internal ions, or even known‐unknown peaks). In this section, we will concentrate on the backbone‐only approach, as it remains the current standard in the field. However, it is important to note that recent advancements, such as the *PredFull* [92] model, have explored full‐spectrum predictions. This expanded approach holds promise for capturing a broader range of fragmentation patterns.

**FIGURE 9:**
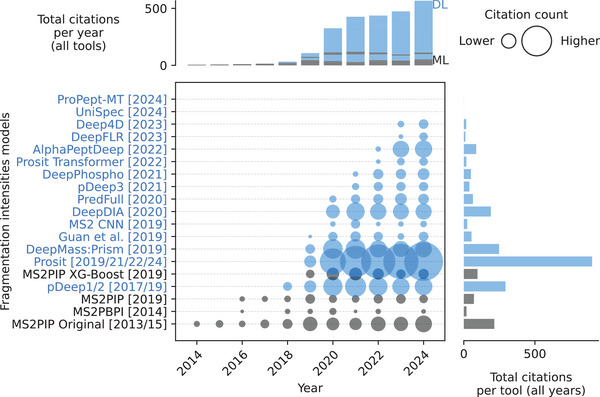
Citations over time for different fragmentation ion intensity models. As of February 18, 2025. The cutoff years are 2010 and 2024. *MS2PIP* Original includes publications from 2013 to 2015. *Prosit* includes publications from 2019, 2021, 2022, 2024, and 2024 (preprint). *pDeep* includes publications from 2017 and 2019. References to all models can be found in Table S1. Models using deep learning (DL) are blue. Models using only classical machine learning (ML) are gray. The exact values of the citation counts per tool and year can be found in Table S2. Created with: https://github.com/jesseangelis/Citation_vis/ and *OpenAlex* [32].

The field of fragmentation ion intensity prediction for peptides (Figure 9 and Table S1) has experienced significant advancements, particularly with the transition from traditional ML methods to deep learning techniques. Early models, such as *MS2PBPI* [93], employ gradient boosting, while more recent models have adopted various neural network architectures, notably BiLSTMs in models like *pDeep3* [94] and *DeepMass:Prism* [44]. The latest trend is the integration of transformer architectures, as seen in *AlphaPeptDeep*, with some models employing hybrid approaches that combine different architectures. The evolution of these models reflects a broader trend in bioinformatics. Input types remain consistent across these models, typically requiring peptide sequence and CS, with some incorporating additional parameters such as collision energy. The choice of loss functions varies, with common options including PCC and MSE. While there is no universally accepted training dataset, many models are trained on in‐house datasets or utilize publicly available resources like *ProteomeTools* [52]. Like with other properties there is a clear preference towards open‐source development, most licensed under Apache‐2.0 and available on platforms like *GitHub*, supplying both training and inference code. Citation trends indicate a dynamic field with continuous innovation. Early traditional ML models gradually increased in citations, while DL models introduced around 2019 saw significantly rising interest. Models like *Prosit* and *MS2PIP* have maintained high citation counts, reflecting their foundational impact on the field, while newer models such as *AlphaPeptDeep* have quickly gained traction. However, it is important to note that both *Prosit* and *AlphaPeptDeep* are also used to predict other properties, which likely skews the citation counts.

#### 2.5.2 Introduction to *Prosit*

**FIGURE 10:**
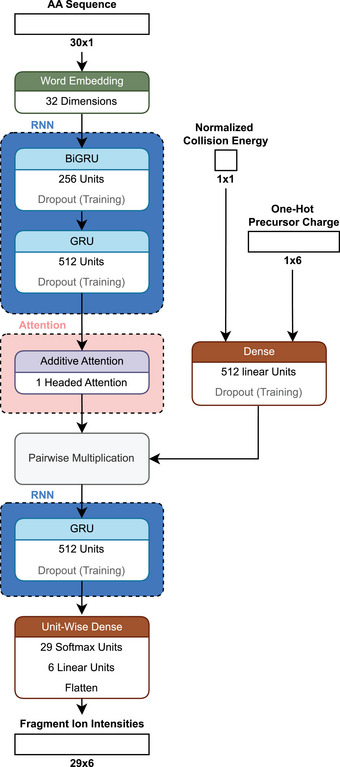
Schematic architecture of *Prosit* [51].

The *Prosit* model [51] is among the most used peptide property prediction models today. The base version of *Prosit* was trained on a large, publicly available dataset generated as part of the *ProteomeTools* project [52] that contained 331,000 synthetic peptides and 5.43 million peptide‐spectrum matches. It encodes the amino acid sequence by word embeddings. In addition, the one‐hot encoded charge state of the peptide ion of interest and a value for the normalized collision energy are fed into a dense neural network (Figure 10). The word embedding is transformed by two stacked GRUs, allowing it to capture the context of the sequence when going along each amino acid. In the original publication, all GRUs are described as bidirectional. However, the model files suggest that only the first GRU layer is a BIGRU [51].

The information from the GRU layers is then given to an attention layer. The attention mechanism is crucial to the success of the transformer architecture, which has driven the development of powerful large language models like the once behind *ChatGPT*. In an attention layer, the relative importance of one element to another is evaluated, contrasting sharply with the sequential nature of LSTMs and GRUs. Through sophisticated matrix calculations, a value matrix is generated, indicating how much one value should attend to (or be influenced by) another. These attention values are then used to update the original embeddings, ensuring that primarily relevant information guides the update process [95]. This explanation, however, merely scratches the surface of the topic, and a full explanation is out of the scope for our review. For those interested in a deeper understanding of attention, we recommend exploring the visualizations provided by the 3blue1brown YouTube channel [96]. While not peer reviewed, a visual grasp of the underlying matrix calculations might help newcomers navigate the technical peer‐reviewed papers more easily, lowering the barrier to entry in this field.

Continuing along the *Prosit* architecture, the processed sequence information is pairwise multiplicated with information on the normalized collision energy and precursor charge to form a single latent space [51]. Latent space refers to a multi‐dimensional representation of data where complex features and patterns are captured in a simplified vectorized form [97]. It is decoded by another GRU and a uni‐twise dense network to a matrix representing the intensities of the 174 supported fragments, covering singly, doubly, and triply charged b‐ and y‐ions for peptides up to 30 amino acids. Currently, the base model of *Prosit* only supports carbamidomethylated cysteine and oxidation of methionine as modifications. However, this limitation is envisioned to be removed in the future [51].

For training, a normalized spectral contrast loss was used. This function takes two vectors representing the true spectrum $V_{a}$ and the prediction $V_{b}$. These vectors are L2 normalized, turning them into unit vectors. Then the angle between them is calculated by applying the arccos. This angle is normalized to result in a value of 0 if the vectors align, 1 if they are orthogonal or 2 if they are antiparallel:

(2) $$L=\frac{2\cos^{-1}\left(V_{a}\cdot V_{b}\right)}{\pi }$$

A recent study [91] compared different deep learning fragmentation prediction models. It was found that *Prosit* outperforms all other models except for when predictions are made for measurements done on a QExactive instrument using HCD. In this case, *Prosit* is recommended to be either finetuned or replaced with *pDeep3* or *AlphaPeptDeep*. However, since a QExactive instrument is also using HCD (beam‐type CID) for fragmentation, it may be reasonable to assume that the difference in prediction performance is largely a result of differences in the mass spectrometers (e.g., ion transfer efficiencies) which may result in slight overfitting of the model, rather than a per se difference in prediction performance with respect to the fragmentation method. In the comparison of the different models their published versions were used. In some cases, they were fine‐tuned but it was decided to not retrain them from scratch [91]. For a meaningful comparison of architecture and loss function choices, however, common benchmark datasets and clearly defined evaluation criteria are essential. Unfortunately, this issue is not limited to fragmentation prediction alone. To our knowledge, none of the properties discussed above have community‐wide accepted standards for quantifying model performance. This lack hampers model comparability, making it challenging for researchers to identify areas for improvement and to apply successful approaches from one property to another.

In contrast to *Prosit*’s normalized spectral contrast loss, *pDeep3* uses the PCC to assess the similarity between spectra and *AlphaPeptDeep* implements an L1 loss [47, 94]. These three loss functions represent different learning approaches. The spectral contrast loss in theory focuses on the general shape of the spectrum, penalizing spectra with poor relative intensities compared to their ground truths. Furthermore, being a measure based on angles in the vector space it is invariant to the actual scale of the values. L1 loss instead focuses on the actual difference in intensity values, which in the case of *AlphaPeptDeep* were normalized beforehand, as it is sensitive to different scales [47]. The PCC captures the linear relationship between the spectra, making it sensitive to the overall trend, but it does not account for small variations in ratios as effectively as the spectral angle [98], placing it between spectral contrast loss and L1 loss. Given a community wide accepted benchmark dataset, it would be interesting to see how each architecture performs when trained with the different loss functions, to investigate whether the focus on spectrum shape is indeed a factor for driving model performance.

#### 2.5.3 Limitations and Future Direction

An, to the best of our knowledge, overlooked aspect when talking about fragmentation spectrum prediction is that while the training data will also contain a small fraction of false positive peptide spectrum matches (PSMs) (≤ 1% FDR), the FDR at the fragment level, the information the model is trained on, is essentially unknown. Even high‐scoring true positive PSMs are likely to contain incorrectly annotated peaks. Depending on the intensity of these incorrect peaks, the losses used to train a model can be thrown off significantly. This may also explain the long “tails” typically observable in performance distributions.

DL models have improved peptide fragment ion intensity prediction in tandem MS. However, challenges persist, such as limited handling of diverse PTMs, difficulty in generalizing across instruments and fragmentation methods, and a lack of standardized benchmarks for consistent model evaluation. Loss functions vary across models with *Prosit*’s spectral contrast loss, *AlphaPeptDeep*’s L1 loss, and *pDeep3*’s PCC, each emphasizing different facets of spectral similarity. The likely presence of false positive PSMs in training data, combined with poorly defined false discovery rates at the fragment level, adds further complexity. Addressing these limitations could significantly enhance the robustness and applicability of fragmentation prediction models in proteomics.

### 2.6 Detectability

#### 2.6.1 Background

**FIGURE 11:**
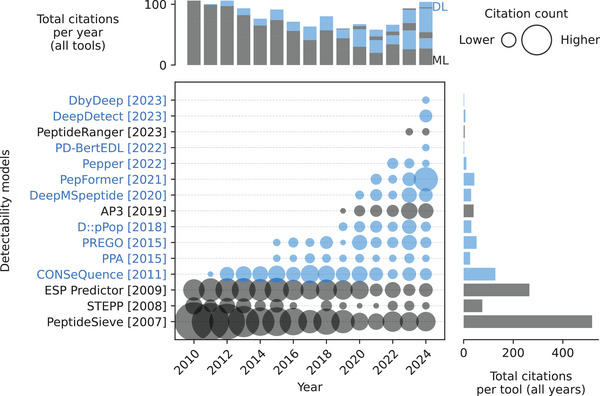
Citations over time for different detectability (flyability) models. As of February 18, 2025. The cutoff years are 2010 and 2024. *Pepper* is the only flyability model. References to all models can be found in Table S1. Models using deep learning (DL) are blue. Models using only classical machine learning (ML) are gray. The exact values of the citation counts per tool and year can be found in Table S2. Created with: https://github.com/jesseangelis/Citation_vis/ and *OpenAlex* [32].

While the aforementioned properties all are important for the accurate detection and quantification of previously unmeasured peptides and thus proteins, one could view the problem from a more general perspective, asking if a peptide is typically present when investigating a certain protein (proteotypicity), is it able to ionize and travel along the mass spectrometer (flyability), is it then also detectable (detectability), and how well its abundance also represents the parent‐protein's abundance (quantotypicity). These properties are usually discussed together or even used synonymously as they are inadvertently connected to each other and many models have been published aiming to predict them (Figure 11 and Table S1). A peptide can only be quantotypical for a protein if it is proteotypic, able to fly, and accurately detectable. For that, it must be present after digestion and fragment well to be identified consistently. Therefore, detectability and more over quantotypicity should be considered more relevant properties of peptides. Especially quantotypicity includes all other properties like digestibility and flyability. Due to the physical limitations of current mass spectrometers only a subset of peptides can be accurately investigated by MS2 within a reasonable time. Thus, knowledge about the most quantotypical peptides is important to adjust the search space accordingly [91, 92].

The models proposed to predict detectability typically use the peptide sequence as input, and in some cases, they also incorporate calculated physicochemical properties. Like with other properties, there has been a shift toward more complex DL models around 2020. Initially, CNNs were the preferred architecture, but current trends have favored sequence‐oriented architectures such as BiLSTMs and transformers. Commonly used loss functions for detectability models include binary cross‐entropy (BCE) and MSE. While earlier models such as *PeptideSive* [99] and *ESP Predictor* [100] have seen a decline in popularity, as indicated by decreasing citation counts, newer models like *PepFormer* [101] and *DeepDetect* [102] are gaining traction.

#### 2.6.2 Introduction to *DeepDetect*

**FIGURE 12:**
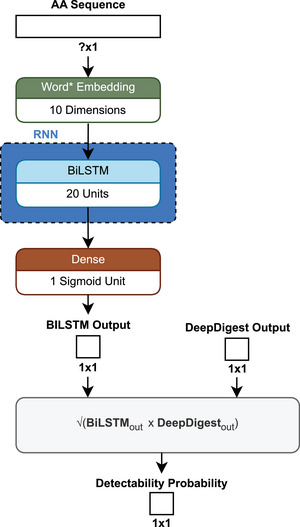
Schematic architecture of *DeepDetect* [102]. *Not clearly defined in the publication but assumed to be word embedding.

The authors of the previously discussed *DeepDigest* [25] considered digestion as a significant factor for the prediction of proteotypic peptides, because it directly influences the availability of peptides for subsequent analysis and identification in an MS experiment. They used *DeepDigest*’s digestibility probabilities in combination with another probability predicted by a simple BiLSTM to get a detectability probability, to build a new model called *DeepDetect* [102] (Figure 12). The publication does not define the maximum sequence length nor the actual type of embedding. *DeepDetect* was trained with a BCE loss function to assess whether a given peptide is detectable in the actual measurement. The addition of the digestion probability to the simple BiLSTM showed an increase in the AUC from 0.721–0.949 to 0.845–0.976 depending on the protease used. Further, the digestion probability was added to one of the previous state of the art models, *PepFormer*, where it increased the AUC from 0.682–0.903 to 0.825–0.972 depending on the protease [102].

Predictions made by *DeepDetect* were used to filter existent spectral libraries, which were then used to detect proteins in plasma and yeast samples. Searching for only the top 40% of peptides based on detectability decreased the runtime by 42.6% for plasma and 14.8% for yeast, while increasing the precursor identifications by 0.9% and 2.0%, as well as the protein group identifications by 3.6% and 0.3%, respectively [102].

#### 2.6.3 Introduction to *PepFormer*

**FIGURE 13:**
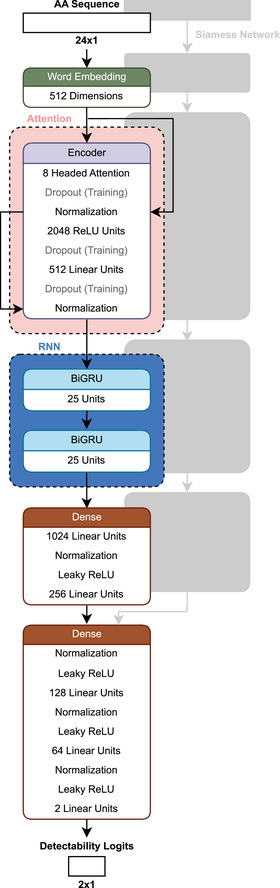
Schematic architecture of *PepFormer* [101].

*PepFormer* takes a unique approach to detectability predictions by employing a Siamese network (Figure 13). This architecture aims to generate similar vector representations for peptides with comparable detectabilities. Specifically, a Siamese network processes two different peptides through identical weights, yielding two outputs that are subsequently compared to assess their similarity. For training, the authors applied contrastive loss as follows:

(3) $$L\left(v_{1},v_{2},Y\right)=\frac{1}{2}\left(1-Y\right)D(v_{1},v_{2})2+\frac{1}{2}Y(\max\left(0,m-D(v_{1},v_{2})2\right)$$

where **v**_1_ and **v**_2_ are the two vector representations for each peptide, *D* (**v**_1_, **v**_2_) is their Euclidean distance, *m* is a margin parameter, and Y is a binary indicator: Y=0 if both peptides are either detectable or undetectable, and Y=1 if they differ. Additionally, for each vector representation, an independent cross‐entropy loss was computed for the numerical value of the predicted detectability of each peptide [101].

#### 2.6.4 Introduction to *Pepper*

**FIGURE 14:**
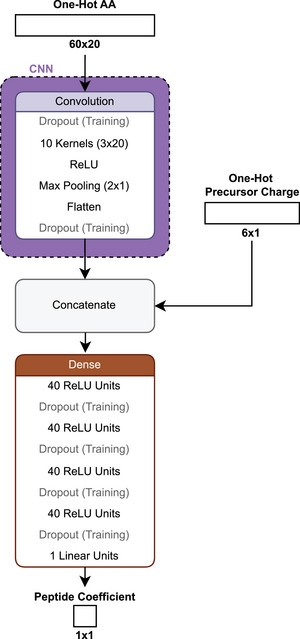
Schematic architecture of *Pepper* [103].

A recent model that was proposed to quantify biases associated with flyability is *Pepper* [103]. It follows the assumption that peptides stemming from the same parent protein have the same abundance and that their intensities could be quantified as a sequence dependent coefficient multiplied with the adjusted observed parent protein's abundance. *Pepper* aims to predict these coefficients. To verify the first assumption, multiple sibling peptide pairs were selected, and their observed quantity ratios across different runs were compared. Specifically, the ratio of a peptide in two runs was evaluated against the corresponding ratio of its sibling peptide in the same runs. While the PCC of 0.39 was considered high and used to prove that sibling pairs have equal abundances [103], this correlation should be considered only moderate. Therefore, indicating that if the assumption is true, the peptide‐specific bias is at least not linear. To prove that Pepper reduces this bias for protein quantification, it was shown that the correlation between this adjusted protein quantification and the respective mRNA expression was increased. However, this increase was rather small with a ∼0.03 Pearson correlation, while the absolute Pearson correlation was in the range of ∼0.3 to ∼0.46 which is only moderate at max [103]. This is likely due to the many factors influencing the abundance of a protein outside of mRNA presence like proteolysis or translation rate. While the approach *Pepper* takes is interesting, there is still more research needed to quantify flyability. In Pepper's architecture (Figure 14), an unusual design choice is the placement of a dropout layer immediately after the one‐hot encoding of the peptide sequence. Dropout, which randomly sets values to zero during training to prevent overfitting, is typically not applied to inputs or one‐hot encoded values, as they already contain mostly zeros. If the layer sets the single “1” in the encoding to zero, it effectively simulates a missing amino acid, or “uncertainty” on the input sequence. The impact of this approach on model performance remains unclear but could worsen model performance substantially.

#### 2.6.5 Limitations and Future Direction

As mentioned above, both *PepFormer* and *DeepDetect* exhibit comparable performance when digestibility predictions are incorporated [102]. This similarity suggests that model architecture alone may not significantly influence predictive capabilities. Instead, meaningful input features, such as digestibility, are likely the primary determinants of model performance. However, it is worth questioning whether *DeepDigest* does in fact predict digestibility or if it is already a detectability predictor. In its training data, only peptides that were both digested and detected are labeled as positive examples. Peptides that were not detected are assumed to be not digested, which introduces a bias toward detectable peptides. In clearer terms, providing any model with the results it is to predict will likely result in good performance. To predict upstream properties like digestability the training data must not be influenced by any downstream property like flyability or detectability or at least correct for these factors.

While it is questionable whether *DeepDetect* has actually utilized this approach, the concept of building stacked or hierarchical models remains largely unexplored in MS‐based proteomics. To our knowledge, no viable model currently exists for quantotypicity prediction. However, developing such a model would likely require robust predictions of upstream features. Ideally, such a model could learn directly from the training data. In practice, though, the complexity of quantotypicity poses challenges, as the training data may lack sufficient sample diversity, contain high levels of noise, or have an unbalanced representation of critical peptide features. These limitations might hinder a model's ability to capture all relevant parameters without additional prior information in the form of predictions. In a larger scope, integrating multiple predictive models into a comprehensive framework could enhance data quality estimation and provide ground‐truth data for evaluating search algorithms [12].

In alignment with *DeepDigest*, *DeepDetect* does not allow for the incorporation of PTMs [25, 102]. The same is also true for *PepFormer* [101]. This limitation as discussed earlier is a desirable point of improvement for future models. Detectability, quantotypicity, and flyability are essential properties that build upon many of the previously discussed peptide characteristics. However, while detectability can be predicted reasonably well by incorporating the digestibility predictor *DeepDigest*, as done in *DeepDetect*, to our knowledge, no effective models currently exist for predicting flyability or quantotypicity. Further work is needed, particularly to expand models to include modified peptides. The promising approach of incorporating predictors of upstream properties may pave the way for a viable quantotypicity predictor.

## 3 Related Peptide Property Prediction

We have discussed key peptide properties, including digestibility, retention time, charge states, collisional cross section in ion mobility, fragmentation ion intensities, flyability, detectability, and quantotypicity. However, many additional peptide properties, like surface area, gas‐phase basicity, flexibility or rigidity, stability, and synthesizability, play significant roles in molecular behavior but lie beyond the scope of this review. In the following sections, we highlight two specific properties, hydrophobicity and 3D structure, which are particularly relevant to MS‐based proteomics due to their substantial influence on molecular behavior in general.

### 3.1 Hydrophobicity

#### 3.1.1 Background

Hydrophobicity is a key property of peptides that directly and indirectly (through protein structure) influences various steps in an MS‐based workflow, such as digestibility, chromatographic behavior, and ionization efficiency [104, 105, 106]. Traditionally, the hydrophobicity of peptides is estimated by applying experimentally derived hydrophobicity scales based on amino acid sequences [107, 108]. However, hydrophobicity should be understood as a broad concept rather than a single numeric value. One widely used measure of hydrophobicity is the octanol‐water partition coefficient (KOW or log *p*). The traditional method for experimentally determining log *p* is the shake flask method. Here, a compound is dissolved in both octanol and water, shaken until equilibrium is reached, and the concentration in each solvent is measured. The ratio of the compound's concentration in octanol to water is calculated, and the logarithm of this value gives log *p*. Positive log *p* values indicate hydrophobic compounds, while negative values indicate hydrophilic compounds [109, 110, 111, 112]. Another measure, log *D*, considers the compound's distribution between its charged and uncharged forms across different pH levels. Log *D* provides a more comprehensive understanding of how a compound behaves in both its ionized and unionized states, which is important in biological environments and especially for peptides [113, 114].

#### 3.1.2 State of the Art

**FIGURE 15:**
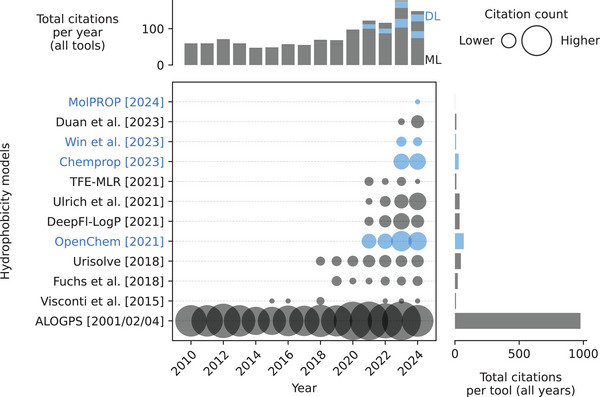
Citations over time for different hydrophobicity models. As of February 18, 2025. The cutoff years are 2010 and 2024. *ALOGPS* includes publications from 2001, 2001, 2002, 2004, and 2004. References to all models can be found in Table S1. Models using deep learning (DL) are blue. Models using only classical machine learning (ML) are gray. The exact values of the citation counts per tool and year can be found in Table S2. Created with: https://github.com/jesseangelis/Citation_vis/ and *OpenAlex* [32].

There has been a substantial amount of research aiming to predict hydrophobicity of compounds (Table S1). In fact, most citations regarding hydrophobicity prediction models in general these days still go to one of the original models in this field, *ALOGPS* [115, 116, 117, 118, 119] (Figure 15). The prediction of hydrophobicity for peptides specifically has not been widely explored, with only a handful of studies [120, 121, 122]. These studies are hindered by small datasets (fewer than 1000 samples) and, because of this, rely on traditional ML techniques and do not use more advanced DL models. The existing models focusing on peptide hydrophobicity demonstrate notable weaknesses in their predictions. One study, Visconti et al. [122], observed *R* ^2^ values below 0.91, indicating only moderate predictive accuracy, while another, Fuchs et al. [121], reported an RMSE of 0.6, which is significantly higher than the experimental standard deviation of 0.08. This suggests that the model's prediction errors exceed the natural variability found in experimental data. Notably, in the latter study, 75% of the test data were also used for training, which likely led to an overestimation of model performance due to data leakage (see Supporting Information). When the same model was tested on an in‐house dataset of 15 peptides, it had an RMSE of 0.9, with 46.7% of predictions showing errors greater than 1.0 log units when compared to actual measurements [121].

Besides its impact on peptides in MS experiments, hydrophobicity is also a critical factor in drug development (here mostly referred to as lipophilicity), as it greatly influences a drug's mode of action and how well it is absorbed by the body [123]. Thus most models that aim to predict hydrophobicity are developed from a drug development perspective. A recent review [124] on in silico drug absorption provides an extensive overview of models used for predicting log *p* and log *D*. For a detailed list of these models, refer to that review. The log *D* prediction models presented there show comparable results to the peptide specific ones discussed earlier, with reported *R* ^2^ values around 0.9 and RMSEs of approximately 0.5 [124]. However, most of these models are primarily trained on datasets of drug‐like molecules, which may include only a limited number of peptides or peptide derivatives. As a result, predicting properties like hydrophobicity or lipophilicity for peptides could fall outside the training distribution of these models. To determine their accuracy in peptide‐specific predictions, a benchmarking study would be necessary to evaluate their performance in predicting peptide hydrophobicity.

#### 3.1.3 Limitations and Future Direction

A key challenge in creating a suitable benchmarking dataset is ensuring a high quality of data. One study [125] identified several issues in the datasets they analyzed, such as incorrect log *p* values due to transcription errors or improper conversions. Other problems included incorrect molecular identifiers or SMILES notations, unsuitable experimental conditions for determining log *p*, and inherent measurement errors. Even under ideal conditions, these errors can range between 0.2 and 0.4 log *p* units, which affects both the reliability of the data, and the models trained on it [125].

Given that hydrophobicity is a key property influencing other properties such as digestion and retention time [48, 126], it is likely that more interest and research in this area will grow, especially if large datasets become available. The peptide specific studies mentioned also lack publicly available code or usable models, which limit their accessibility and reproducibility [121, 122]. Beyond the data limitations, a recent review [120] highlighted another issue. Most studies only consider octanol as the solvent, which could overlook the effects of conformational changes that peptides undergo in different environments. Additionally, they pointed out that no current study incorporates 3D structural information, which becomes crucial when peptides form secondary structures. This missing aspect could be a significant factor in accurately predicting peptide properties [120].

### 3.2 3D Structure

#### 3.2.1 Background

**FIGURE 16:**
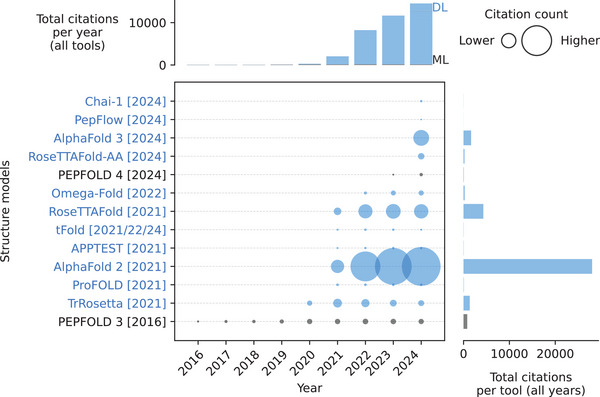
Citations over time for different 3D structure models. As of February 18, 2025. The cutoff years are 2010 and 2024. *tFold* includes publications from 2021, 2022 (preprint), and 2024 (preprint). *Chai‐1* is a preprint. *RoseTTAFold* includes two publications from 2021. *Omega‐Fold* is a preprint. References to all models can be found in Table S1. Models using deep learning (DL) are blue. Models using only classical machine learning (ML) are gray. The exact values of the citation counts per tool and year can be found in Table S2. Created with: https://github.com/jesseangelis/Citation_vis/ and *OpenAlex* [32].

Structural knowledge of proteins and peptides is crucial for various areas in biology, like drug development, where it helps to improve their therapeutic potential by improving solubility, absorption, and permeability [127]. However, determining these 3D structures is a non‐trivial task due to the influence of various environmental factors, such as pH and temperature, on peptide folding [128]. The field of protein structure prediction has enjoyed a lot of interest with many models being proposed over the time (Figure 16 and Table S1).

#### 3.2.2 State of the Art

Given the significance and difficulty of this problem, it is, therefore, no surprise that Google DeepMinds' *AlphaFold2* [129] made headlines beyond academia with its remarkable performance in the 2020 protein folding assessment, CASP14, displaying a substantial leap in predictive accuracy [130, 131, 132]. Most notably two key figures behind *AlphaFold2* were awarded the Nobel Prize in chemistry [10]. However, when talking about *AlphaFold2* it is important to keep in mind that CASP14 evaluates predictions for protein crystal structures, not peptides, and not all targets were successfully predicted [130]. Two years after *AlphaFold2*’s release, its capabilities in peptide structure prediction were examined [128]. It was found that *AlphaFold2*’s predictions closely matched experimentally determined structures, particularly when the peptides had well‐defined secondary structures without multiple turns. It was also highlighted that the inherent flexibility of peptides complicates accurate structure prediction, as conformations may vary with specific experimental conditions like pH or temperature. Furthermore, *AlphaFold2* might predict alternative low‐energy conformations that differ from those determined experimentally. The study compared *AlphaFold2* to other structure prediction models, including *PEP‐FOLD3* [133], *Omega‐Fold* [134], *RoseTTAFold* [135], and *APPTEST* [136], demonstrating that *AlphaFold2* either outperformed or was at least comparable to these alternatives [128].

Since then, *AlphaFold* has been updated to *AlphaFold3* [137], which focuses on predicting the structures of complexes. It features significantly different architecture. *AlphaFold2* uses an “evoformer,” which is a type of network that aims to extract information about evolutionary conserved regions that could be important for the structure. It then predicted relative angles between atoms based on this information [129]. In contrast, *AlphaFold3* places less emphasis on evolutionary data, employing a technique called diffusion to generate atom positions [137]. This approach was previously explored in *RoseTTAFold All‐Atom* [138]. Diffusion models, known for their application in high‐quality image generation (e.g., *StableDiffusion* [2]), can be used to denoise noisy data/pictures. If they are supplied with random noise, they will instead generate a completely new image [139]. Similarly, *AlphaFold3* starts with random atom coordinates and feeds information about the molecules into the diffusion module to derive the final structure through recurrent denoising [137].

*AlphaFold3*’s architecture consists of different modules that each have a specific task. The input encoder converts unstandardized inputs into a computationally usable array format. The template and multi‐sequence alignment modules incorporate information from structurally similar templates and genetically similar sequences, respectively. The “pairformer” then changes the information‐loaded arrays to represent more abstract features of the input. The diffusion module performs diffusion on random atom positions to infer the final structure [137].

During training, different losses are calculated based on the performance of each module and then combined to the final loss function:

(4) $$\begin{array}{ccc} L_{\mathrm{loss}} & = & \alpha_{\mathrm{confidence}}\cdot \left(L_{\mathrm{pLDDT}}+L_{\mathrm{PDE}}+L_{\mathrm{resolved}}+\alpha_{\mathrm{PAE}}\cdot L_{\mathrm{PAE}}\right)\\ & & +\alpha_{\mathrm{diffusion}}\cdot L_{\mathrm{diffusion}}+\alpha_{\mathrm{distogram}}\cdot L_{\mathrm{distorgram}} \end{array}$$

Here, the different loss terms are weighted with $\alpha_{\mathrm{confidence}}$ = 10^−4^, $\alpha_{\mathrm{diffusion}}$ = 4, and $\alpha_{\mathrm{distogram}}$ = 3 ×·10^−2^. The training process consists of one initial training phase followed by three fine‐tuning steps. *AlphaFold* predicts multiple confidence metrics. $\alpha_{\mathrm{PAE}}$ is always zero except for the last step of fine‐tuning where it becomes one [123]. The local distance difference test (LDDT) measures how well local structures are represented in the 3D‐model. *AlphaFold* tries to predict this measurement with the predicted local distance difference test (pLDDT). This involves estimating the probabilities of each atom's LDDT confidence measurement falling into bins spanning from 0 to 1. Knowing the correct confidence that each atom is supposed to fall into, the loss is calculated using cross‐entropy, which is punishing low probabilities for the correct bins. This is done by calculating the negative log of the respective probability. The same approach is used to calculate the loss on the predicted alignment error L_PAE_, the loss on predicted distance error $L_{\mathrm{PDE}}$, and the loss on the prediction whether a given atom would be resolved in an experimentally determined structure $L_{\mathrm{resolved}}$. The predicted alignment error (PAE) estimates how well the model would align to an actual measurement. The predicted distance error (PDE) estimates the absolute distances between model atoms and alignment atoms [137]. To our knowledge confidence estimation is underutilized in MS‐based proteomics models. Implementing a learned confidence measure could enable future models to focus on simpler examples initially, progressing to more challenging ones as their confidence builds. Cross entropy was also used to predict the loss of the distrogram $L_{\mathrm{distorgram}}$, which captures pairwise distances of molecular features like protein residues, purines, and pyrimidines [137]. The diffusion module loss $L_{\mathrm{diffusion}}$ consists of the mean square distance of all model atoms to their ground truth counterpart, the bond length error, which is the mean squared difference of bond distances between model and ground truth, and a version of LDDT [137].

#### 3.2.3 Limitations and Future Direction

By their fundamental mechanism, diffusion models are prone to hallucination, meaning that they produce solutions that are not based on real‐world data. While this capacity is advantageous in image generation, it poses challenges for predicting biochemical structures governed by the laws of physics. In *AlphaFold3*, for example, stereochemistry violations or unstructured areas that lack the ribbon‐like patterns found in many biomolecules were observed. Even with a term added to penalize stereochemistry violations when ranking different predictions, they encountered these violations 4.4% of the times [137]. In contrast, *RosettaFold All‐Atom* uses loss terms that explicitly addressed stereochemistry violations, resulting in predominantly stereochemically sound structures, although numerical values for this assumption were not provided in the study [138]. To evaluate the impact of these specialized loss terms on stereochemical accuracy, further research is necessary.

In the *AlphaFold3* publication, a significant limitation of current structure prediction models was acknowledged. They are trained to predict static structures, while real molecules exhibit flexibility in solution [137]. This limitation is particularly critical for predicting highly flexible peptide structures. Additionally, it is essential to recognize that *AlphaFold* only determines final structures; it does not provide a comprehensive explanation of the folding process. While *AlphaFold3* has demonstrated increased accuracy for the structure predictions of most biological complexes [137], it remains an open question how its peptide structure prediction capabilities compare to those of *AlphaFold2*.

Open Sourcing a model is vital for the scientific community to test and build upon previous work. However, in highly competitive fields like drug discovery, this openness is often not possible, limiting the usability of state‐of‐the‐art models. Until recently the *AlphaFold3* model was not made available open source, prompting other groups to recreate it according to the associated literature. For example, an open source‐model called *Chai‐1* [140] by the company Chai‐Discovery was recently published as a preprint. While it is important to note that there has not been a peer‐reviewed publication yet, the development team wrote that the “… model architecture and training strategy largely follows that of {*AlphaFold3*} …” [140], indicating that it might be a rebuild of *Alphafold3*. The team, who partnered with another big tech firm, OpenAI, compared *Chai‐1* against *AlphaFold3* and reported competitive results [140].

One thing to keep in mind is that the state‐of‐the‐art models for protein structures still have limited applicability for predicting the structures of shorter peptides, which are common in MS‐based proteomics, as they still need validation for these types of peptides. One challenge in predicting their 3D structures is that small peptides are thought to be highly flexible, making their structural determination difficult. This flexibility might also explain why, to our knowledge, no peptide property prediction tools in MS currently leverage 3D structural data. While complete 3D structures of peptides may be elusive, the known molecular structures of individual amino acids, both modified and unmodified, remain largely untapped as well. Incorporating this structural information could provide an adaptable approach to modeling modified peptides, allowing for a more informative input beyond simple atom composition. This approach might enhance the model's generalization capabilities, as atom compositions alone seemingly have limited value in capturing the nuanced structural variations that affect peptide behavior.

## 4 Frameworks

As mentioned in the introduction, this review seeks to introduce newcomers to AI models for peptide property prediction, aiming to lower the barrier to entry in this field. To achieve our goal, we will highlight several frameworks to help new talent quickly develop productive models and explore any ideas inspired by the previous sections. These frameworks enable researchers to build new models with ease. We will focus on frameworks that are built for Python, as this is the de facto standard for machine learning nowadays, and often the first programming language learned with tons of information and courses online [141, 142, 143]. There are several general‐purpose packages for developing deep learning models. These include *Keras* [144], *PyTorch* [145], and *TensorFlow* [146], while *PyTorch* has seen an increase in popularity over the recent years (Table S1).

*Keras* offers a user‐friendly API for rapid prototyping, making it ideal for beginners, while *TensorFlow* excels in production deployment and optimization capabilities. *PyTorch* has gained significant traction in the scientific community due to its flexibility and dynamic computation graph, which facilitate intuitive model development and debugging. Building on *PyTorch* the already mentioned *AlphaPeptDeep* is a framework specifically for peptide property prediction models. While conserving the flexibility of *PyTorch*, it adds a peptide embedder, integration of the *HuggingFace* [147] transformer library, several pre‐built models, and other features. The peptide embedder works by one‐hot‐encoding the amino acids. Modifications are encoded by their chemical composition similar to the approaches of *DeepLC* and *CPred* [47, 48, 63]. Pretrained models are available for using them as bases for transfer learning [47]. Other framework related projects include *DepthCharge* [148], providing MS‐specific *PyTorch* modules and *MS2PIP* [149], which includes some useful functions for peptide property predictions. To track the model along the training and fine‐tuning process, *Weights & Biases* [150] could be used, which provides logging capabilities and visualizations making it easier to monitor a model's process. To store models and training data *HuggingFace* [147] is a popular choice, as it provides structured information about the respective models and datasets. It also offers tutorials, model hosting, and more. An MS‐specialized approach can be found in *ProteomicsML* [151], where community curated datasets and tutorials are provided. Making new models available for the research community is essential to foster discussion and further development. *DLOmix* [152] provides a platform where models can be implemented to be retrained on different or new data in order to benchmark them against other models. This implementation would also allow for easier development of stacked models, combining research efforts instead of developing new models in parallel and not benefiting from independent discoveries on how to best normalize data, which architecture works well, or which loss function alleviates biases best.

## 5 General Limitations and Future Directions

Even though there are many models showing good performances when predicting peptide properties, there are key limitations to doing so. As mentioned in the section on detectability, properties influence each other. This can be downstream (e.g., a peptide must be digestible to be detected) but it can also be upstream (e.g., only accurately detected charge states can be investigated). This means that with the current experimental methods some properties might not yet be predictable in isolation. Missing knowledge about the flyability of a peptide hinders any predictions made for ionization or digestion. In other terms, while our predictions may be accurate for the systems we study, we do not know if they are true.

Models often struggle with generalizing to unknown modifications of amino acids. While there is a common belief that embedding modifications in a flexible way automatically allows for generalizability, the results of models that use such approaches like *DeepLC* and *CPred* do not allow for such a conclusion. Usually, the embedding is done by one‐hot‐encoding the unmodified amino acid sequence and adding another embedding of the modified atom composition. It might be the case that a model is able to learn some information by pure association, but to allow for predictions that result from the chemical nature of a peptide, the model would first need to find a suitable representation of these chemical features. Most chemical features arise not from the atoms alone but are the result of how they are structured in a molecule. We know the chemical structures of the amino acids and their modifications, but this information seems to remain underutilized. It could be possible to embed amino acids not just by their arbitrary one‐letter code and atom composition but by their 2D or even 3D structure [153]. Work on graph embedding and chemical structure representation can be found, for example, in proteomics outside of MS [154], in MS‐based metabolomics [155], or in work on enantiomer separation [156]. For more information, we would like to refer to a recent review on graph embeddings and geometric DL [157]. To our knowledge, this has not been done in MS‐based proteomics so far.

Exploring alternative model architectures beyond the commonly used LSTMs, GRUs, CNNs, and attention mechanisms may offer new insights into peptide property prediction. Graph neural networks could better capture molecular relationships by incorporating structural information directly. Transformer‐based models, such as *Prosit‐Transformer* [158] and *AlphaPeptDeep*, have already been applied in this space, but it may also be worthwhile to frame these problems as language modeling tasks, as seen in *PeptideBERT* [159] and *ProteinBERT* [160]. Additionally, diffusion models were demonstrated to be highly effective in structural biology with *AlphaFold3*. They could present new opportunities if adapted for mass spectrometry‐related tasks. Considering these or other approaches could provide fresh perspectives and advance the field beyond current architectures.

Improving generalizability might also be possible by adapting tailored loss functions, instead of, for example, a simple MSE or MAE. Approaches like those of *Chronologer* and *AlphaFold* indicate that sophisticated losses can be beneficial in improving model performance [49, 129, 137]. To rigorously establish the superiority of specific loss functions or strategies, further dedicated research on these advanced topics is needed. However, evaluating models solely on their ability to predict familiar data limits our understanding of their broader utility. Exploring confidence prediction metrics and mechanisms for OOD detection can provide additional insights into a model's reliability and robustness. Confidence measures can highlight areas where predictions may be less reliable, while OOD detection can identify instances where the model encounters data outside its training distribution. These capabilities are particularly critical for understanding the boundaries of models and for comparing their effectiveness beyond standard performance metrics [161, 162]. Confidence in the models' predictions can also arise from making them explainable. Understanding how a model comes to its conclusion can therefore allow a user to estimate if it is fit for purpose. Some studies already apply explainable AI (XAI) (see Supporting Information) methods [70, 163–165].

DL models are usually quite data hungry, with performance improvements usually associated with more data being available. This calls for larger datasets and, maybe even more important, improvement of data quality and heterogeneity [166]. More specifically, these datasets will need to cover as much of the peptide and modification space as possible. Creating such datasets is time‐ and money‐intensive which for many labs will pose a barrier of entry [52, 75]. Alternatively, the development of more efficient models that can match the performance of current models while being trained on less data is also a direction worth investigating. On a similar note, it is important to consider whether DL should be used for every task. Traditional ML techniques like simple regression or decision trees often excel when dealing with well‐defined, low‐dimensional data or structured features, offering computational efficiency, interpretability, and reduced data requirements. Deep learning, while ideal for complex tasks requiring high‐level abstractions can be unnecessarily complex and prone to overfitting for simpler problems. An example can be found in *ionmob* where the initial projection of the CCS is learned with traditional ML techniques [86]. Ultimately, the choice of approach should align with the problem's complexity, data availability, and computational resources, emphasizing that deep learning is powerful but not universally optimal.

## 6 Conclusion

The use of AI‐driven models for peptide property prediction in MS‐based proteomics has advanced the field, enabling more confident peptide identifications. However, these models still face significant challenges, particularly in generalizing to novel peptide modifications, managing class imbalances, and offering limited interpretability of underlying biochemical mechanisms. Future efforts should prioritize expanding datasets, addressing class imbalance, refining feature representations, and establishing benchmarking standards to further enhance model performance. As research continues, we should expect to see long overdue work on generating standardized high‐quality datasets [20, 167, 168], specific benchmarking tools [169], and services to ease the integration of novel models into proteomics workflows [170].

## Conflicts of Interest

M.W. is a founder and shareholder of OmicScouts GmbH and MSAID GmbH with no operational role in either company. The other authors do not have any conflicts of interest.

## Supporting information



Supporting Information

Supporting Information

Supporting Information

## Data Availability

Data sharing is not applicable to this article as no datasets were generated or analyzed during the current study.

## References

[1] OpenAI, "ChatGPT," (2024), https://chatgpt.com/.

[2] stability.ai, "Image‐Models," (2024), https://stability.ai/stable‐image.

[3] Google, "Gemini," (2024), https://gemini.google.com/.

[4] B. Marr , “Why Hybrid AI Is The Next Big Thing In Tech,” Forbes (2024), https://www.forbes.com/sites/bernardmarr/2024/10/02/why‐hybrid‐ai‐is‐the‐next‐big‐thing‐in‐tech/.

[5] Z. Kleinman , “Microsoft: 'ever present' AI Assistants Are Coming,” BBC (2024), https://www.bbc.com/news/articles/czj9vmnlv9zo.

[6] D. B. Catacutan , J. Alexander , A. Arnold , and J. M. Stokes , “Machine Learning in Preclinical Drug Discovery,” Nature Chemical Biology 20, no. 8 (2024): 960–973, 10.1038/s41589-024-01679-1. 39030362

[7] M. K. G. Abbas , A. Rassam , F. Karamshahi , R. Abunora , and M. Abouseada , “The Role of AI in Drug Discovery,” ChemBioChem 25, no. 14 (2024): 2023008, 10.1002/cbic.202300816. 38735845

[8] V. Eyring , W. D. Collins , P. Gentine , et al., “Pushing the Frontiers in Climate Modelling and Analysis with Machine Learning,” Nature Climate Change 14, no. 9 (2024): 916–928, 10.1038/s41558-024-02095-y.

[9] V. Eyring , P. Gentine , G. Camps‐Valls , D. M. Lawrence , and M. Reichstein , “AI‐Empowered Next‐Generation Multiscale Climate Modelling for Mitigation and Adaptation,” Nature Geoscience 17, no. 10 (2024): 963–971, 10.1038/s41561-024-01527-w.

[10] Nobel Prize, “Press Release,” (2024), https://www.nobelprize.org/prizes/chemistry/2024/press‐release/.

[11] B. Wen , W. F. Zeng , Y. Liao , et al., “Deep Learning in Proteomics,” Proteomics 20, no. 21‐22 (2020): 1900335, 10.1002/pmic.201900335. 32939979 PMC7757195

[12] B. A. Neely , V. Dorfer , L. Martens , et al., “Toward an Integrated Machine Learning Model of a Proteomics Experiment,” Journal Proteome Research 22, no. 3 (2023): 681–696, 10.1021/acs.jproteome.2c00711. PMC9990124 36744821

[13] J. G. Meyer , “Deep Learning Neural Network Tools for Proteomics,” Cell Reports Methods 1, no. 2 (2021): 100003, 10.1016/j.crmeth.2021.100003. 35475237 PMC9017218

[14] J. Cox , “Prediction of Peptide Mass Spectral Libraries With Machine Learning,” Nature Biotechnology 41, no. 1 (2023): 33–43, 10.1038/s41587-022-01424-w. 36008611

[15] X. Chen , C. Li , M. T. Bernards , Y. Shi , Q. Shao , and Y. He , “Sequence‐Based Peptide Identification, Generation, and Property Prediction With Deep Learning: A Review,” Molecular Systems Design & Engineering 6, no. 6 (2021): 406–428, 10.1039/D0ME00161A.

[16] O. Bárcenas , C. Pintado‐Grima , K. Sidorczuk, et al., “The Dynamic Landscape of Peptide Activity Prediction,” Computational and Structural Biotechnology Journal 20 (2022): 6526–6533, 10.1016/j.csbj.2022.11.043. 36467580 PMC9712827

[17] Z. L. Chen , P. Z. Mao, W. F. Zeng , H. Chi , and S. M. He , “pDeepXL: MS/MS Spectrum Prediction for Cross‐Linked Peptide Pairs by Deep Learning,” Journal Proteome Research 20, no. 5 (2021): 2570–2582, 10.1021/acs.jproteome.0c01004. 33821641

[18] Y. Yang and Q. Fang , “Prediction of Glycopeptide Fragment Mass Spectra by Deep Learning,” Nature Communications 15, no. 1 (2024): 2448, 10.1038/s41467-024-46771-1. PMC10951270 38503734

[19] O. Shouman , W. Gabriel , V. Giurcoiu , V. Sternlicht , and M. Wilhelm, “PROSPECT,” *Zenodo* (2022), 10.5281/ZENODO.6602020.

[20] B. Wen and W. S. Noble , “A Multi‐Species Benchmark for Training and Validating Mass Spectrometry Proteomics Machine Learning Models,” Scientific Data 11, no. 1 (2024): 1207, 10.1038/s41597-024-04068-4. 39516479 PMC11549408

[21] E. J. Dupree , M. Jayathirtha , H. Yorkey , M. Mihasan , B. A. Petre , and C. C. Darie , “A Critical Review of Bottom‐Up Proteomics: The Good, the Bad, and the Future of this Field,” Proteomes 8, no. 3 (2020): 14, 10.3390/proteomes8030014. 32640657 PMC7564415

[22] M. Kalhor , J. Lapin , M. Picciani , and M. Wilhelm , “Rescoring Peptide Spectrum Matches: Boosting Proteomics Performance by Integrating Peptide Property Predictors Into Peptide Identification,” Molecular & Cellular Proteomics 23, no. 7 (2024):100798, 10.1016/j.mcpro.2024.100798. 38871251 PMC11269915

[23] V. Demichev , C. B. Messner , S. I. Vernardis , K. S. Lilley , and M. Ralser , “DIA‐NN: Neural Networks and Interference Correction Enable Deep Proteome Coverage in High Throughput,” Nature Methods 17, no. 1 (2020): 41–44, 10.1038/s41592-0190638-x. 31768060 PMC6949130

[24] A. F. M. Altelaar , J. Munoz , and A. J. R. Heck , “Next‐Generation Proteomics: Towards an Integrative View of Proteome Dynamics,” Nature Review Genetics 14, no. 1 (2013): 35–48, 10.1038/nrg3356. 23207911

[25] J. Yang , Z. Gao , X. Ren , et al., “DeepDigest: Prediction of Protein Proteolytic Digestion With Deep Learning,” Analytical Chemistry 93, no. 15 (2021): 6094–6103, 10.1021/acs.analchem.0c04704. 33826301

[26] S. Schopper , A. Kahraman , P. Leuenberger , et al., “Measuring Protein Structural Changes on a Proteome‐Wide Scale Using Limited Proteolysis‐Coupled Mass Spectrometry,” Nature Protocols 12, no. 11 (2017): 2391–2410, 10.1038/nprot.2017.100. 29072706

[27] Z. Gao , C. Chang , J. Yang , Y. Zhu , and Y. Fu , “AP3: An Advanced Proteotypic Peptide Predictor for Targeted Proteomics by Incorporating Peptide Digestibility,” Analytical Chemistry 91, no. 13 (2019): 8705–8711, 10.1021/acs.analchem.9b02520. 31247716

[28] D. L. Plubell , L. Käll , B.‐J. Webb‐Robertson , et al., “Putting Humpty Dumpty Back Together Again: What Does Protein Quantification Mean in Bottom‐Up Proteomics?,” Journal of Proteome Research 21, no. 4 (2022): 891–898, 10.1021/acs.jproteome.1c00894. 35220718 PMC8976764

[29] C. Lawless and S. J. Hubbard , “Prediction of Missed Proteolytic Cleavages for the Selection of Surrogate Peptides for Quantitative Proteomics,” OMICS: a Journal of Integrative Biology 16, no. 9 (2012): 449–456, 10.1089/omi.2011.0156. 22804685 PMC3437044

[30] T. Fannes , E. Vandermarliere , L. Schietgat , S. Degroeve , L. Martens , and J. Ramon , “Predicting Tryptic Cleavage From Proteomics Data Using Decision Tree Ensembles,” Journal of Proteome Research 12, no. 5 (2013): 2253–2259, 10.1021/pr4001114. 23517142

[31] F. Teufel , J. C. Refsgaard , C. T. Madsen , et al., “DeepPeptide Predicts Cleaved Peptides in Proteins Using Conditional Random Fields,” Bioinformatics 39, no. 10 (2023): btad616, 10.1093/bioinformatics/btad616. 37812217 PMC10585352

[32] J. Priem , H. Piwowar , and R. Orr , “OpenAlex: A Fully‐Open Index of Scholarly Works, Authors, Venues, Institutions, and Concepts,” *arXiv preprint* (2022): 2205.01833, 10.48550/arXiv.2205.01833.

[33] A. Bhandare , M. Bhide , P. Gokhale , and R. Chandavarkar , “Applications of Convolutional Neural Networks,” International Journal of Computer Science and Information Technologies 7, no. 5 (2016): 2206–2215.

[34] J. A. Loo , H. R. Udseth , R. D. Smith , and J. H. Futrell , “Collisional Effects on the Charge Distribution of Ions from Large Molecules, Formed by Electrospray‐Ionization Mass Spectrometry,” Rapid Communications in Mass Spectrometry 2, no. 10 (1988): 207–210, 10.1002/rcm.1290021006.

[35] K. D. Yamada and K. Kinoshita , “De Novo Profile Generation Based on Sequence Context Specificity With the Long Short‐Term Memory Network,” BMC Bioinformatics 19 (2018): 272, 10.1186/s12859-018-2284-1. 30021530 PMC6052547

[36] L. Tsiatsiani and A. J. R. Heck , “Proteomics Beyond Trypsin,” FEBS Journal 282, no. 14 (2015): 2612–2626, 10.1111/febs.13287. 25823410

[37] L. J. Castro , D. S. Katz , and F. Psomopoulos , "Working Towards Understanding the Role of FAIR for Machine Learning," *DaMaLOS* (2021): ‐1, 10.4126/FRL01-006429415.

[38] S. Fekete , J. L. Veuthey , and D. Guillarme , “New Trends in Reversed‐Phase Liquid Chromatographic Separations of Therapeutic Peptides and Proteins: Theory and Applications,” Journal of Pharmaceutical and Biomedical Analysis 69 (2012): 9–27, 10.1016/j.jpba.2012.03.024. 22475515

[39] A. J. Alpert , K. Petritis , L. Kangas , et al., “Peptide Orientation Affects Selectivity in Ion‐Exchange Chromatography,” Analytical Chemistry 82, no. 12 (2010): 5253–5259, 10.1021/ac100651k. 20481592 PMC2884984

[40] M. Haberger , M. Leiss , A. K. Heidenreich , et al., “Rapid Characterization of Biotherapeutic Proteins by Size‐Exclusion Chromatography Coupled to Native Mass Spectrometry,” Monoclonal Antibodies 8, no. 2 (2016): 331–339, 10.1080/19420862.2015.1122150. 26655595 PMC4966600

[41] J. Lenco , S. Jadeja , D. K. Naplekov , et al., “Reversed‐Phase Liquid Chromatography of Peptides for Bottom‐Up Proteomics: A Tutorial,” Journal Proteome Research 21, no. 12 (2022): 2846–2892, 10.1021/acs.jproteome.2c00407. 36355445

[42] C. W. Tsai , C. I. Liu , Y. C. Chan , H. H. G. Tsai , and R. C. Ruaan , “Study of Conformation Effects on the Retention of Small Peptides in Reversed‐Phase Chromatography by Thermodynamic Analysis and Molecular Dynamics Simulation,” Journal of Physical Chemistry B 114, no. 35 (2010): 11620–11627, 10.1021/jp101846n. 20712332

[43] W. Sun , L. Zhang , R. Yang , C. Shao , Z. Zhang , and Y. Gao , “Improving Peptide Identification Using an Empirical Peptide Retention Time Database,” Rapid Communications in Mass Spectrometry 23, no. 1 (2009): 109–118, 10.1002/rcm.3851. 19065623

[44] R. Bruderer , O. M. Bernhardt , T. Gandhi , and L. Reiter , “High‐Precision iRT Prediction in the Targeted Analysis of Data‐Independent Acquisition and Its Impact on Identification and Quantitation,” Proteomics 16, no. 15‐16 (2016): 2246–2256, 10.1002/pmic.201500488. 27213465 PMC5094550

[45] L. Moruz and L. Käll , “Peptide Retention Time Prediction,” Mass Spectrometry Reviews 36, no. 5 (2017): 615–623, 10.1002/mas.21488. 26799864

[46] C. Escher , L. Reiter , B. Maclean , et al., “Using iRT, a Normalized Retention Time for More Targeted Measurement of Peptides,” Proteomics 12, no. 8 (2012): 1111–1121, 10.1002/pmic.201100463. 22577012 PMC3918884

[47] W.‐F. Zeng , X.‐X. Zhou , S. Willems , et al., “AlphaPeptDeep: A Modular Deep Learning Framework to Predict Peptide Properties for Proteomics,” Nature Communications 13, no. 1 (2022): 7238, 10.1038/s41467-022-34904-3. PMC9700817 36433986

[48] R. Bouwmeester , R. Gabriels , N. Hulstaert , L. Martens , and S. Degroeve , “DeepLC Can Predict Retention Times for Peptides That Carry As‐Yet Unseen Modifications,” Nature Methods 18, no. 11 (2021): 1363–1369, 10.1038/s41592-021-01301-5. 34711972

[49] D. B. Wilburn , A. E. Shannon , V. Spicer , et al., “Deep Learning From Harmonized Peptide Libraries Enables Retention Time Prediction of Diverse Post Translational Modifications,” *BioRxiv* (2023): 2023.05.30.542978, 10.1101/2023.05.30.542978.

[50] C. Ma , Y. Ren , J. Yang , Z. Ren , H. Yang , and S. Liu , “Improved Peptide Retention Time Prediction in Liquid Chromatography Through Deep Learning,” Analytical Chemistry 90, no. 18 (2018): 10881–10888, 10.1021/acs.analchem.8b02386. 30114359

[51] S. Gessulat , T. Schmidt , D. P. Zolg , et al., “Prosit: Proteome‐Wide Prediction of Peptide Tandem Mass Spectra by Deep Learning,” Nature Methods 16, no. 6 (2019): 509–518, 10.1038/s41592-019-0426-7. 31133760

[52] D. P. Zolg , M. Wilhelm , K. Schnatbaum , et al., “Building ProteomeTools Based on a Complete Synthetic Human Proteome,” Nature Methods 14, no. 3 (2017): 259–262, 10.1038/nmeth.4153. 28135259 PMC5868332

[53] V. Gorshkov and F. Kjeldsen , “Exploiting Charge State Distribution to Probe Intramolecular Interactions in Gas‐Phase Phosphopeptides and Enhance Proteomics Analyses,” Analytical Chemistry 96, no. 3 (2024): 1167–1177, 10.1021/acs.analchem.3c04270. 38183295

[54] M. C. Sullards and J. A. Reiter , “Primary and Secondary Locations of Charge Sites in Angiotensin II (M + 2H)2+ Ions Formed by Electrospray Ionization,” Journal of the American Society for Mass Spectrometry 11, no. 1 (2000): 40–53, 10.1016/S1044-0305(99)00115-4. 10631663

[55] J. M. Burkhart , C. Schumbrutzki , S. Wortelkamp , A. Sickmann , and R. P. Zahedi , “Systematic and Quantitative Comparison of Digest Efficiency and Specificity Reveals the Impact of Trypsin Quality on MS‐based Proteomics,” Journal of Proteomics 75, no. 4 (2012): 1454–1462, 10.1016/j.jprot.2011.11.016. 22166745

[56] K. A. Douglass and A. R. Venter , “Predicting the Highest Intensity Ion in Multiple Charging Envelopes Observed for Denatured Proteins During Electrospray Ionization Mass Spectrometry by Inspection of the Amino Acid Sequence,” Analytical Chemistry 85, no. 17 (2013): 8212–8218, 10.1021/ac401245r. 23901825

[57] B. Challen and R. Cramer , “Advances in Ionisation Techniques for Mass Spectrometry‐Based Omics Research,” Proteomics 22, no. 15‐16 (2022): 2100394, 10.1002/pmic.202100394. 35709387

[58] A. M. Kamel , P. R. Brown , and B. Munson , “Effects of Mobile‐Phase Additives, Solution pH, Ionization Constant, and Analyte Concentration on the Sensitivities and Electrospray Ionization Mass Spectra of Nucleoside Antiviral Agents,” Analytical Chemistry 71, no. 24 (1999): 5481–5492, 10.1021/ac9906429. 10624156

[59] S. B. Ficarro , Y. Zhang , Y. Lu , et al., “Improved Electrospray Ionization Efficiency Compensates for Diminished Chromatographic Resolution and Enables Proteomics Analysis of Tyrosine Signaling in Embryonic Stem Cells,” Analytical Chemistry 81, no. 9 (2009): 3440–3447, 10.1021/ac802720e. 19331382

[60] J. G. Meyer and E. A. Komives , “Charge State Coalescence During Electrospray Ionization Improves Peptide Identification by Tandem Mass Spectrometry,” Journal of the American Society for Mass Spectrometry 23, no. 8 (2012): 1390–1399, 10.1007/s13361-012-0404-0. 22610994 PMC6345509

[61] C. R. Mallet , Z. Lu , and J. R. Mazzeo , “A Study of Ion Suppression Effects in Electrospray Ionization From Mobile Phase Additives and Solid‐Phase Extracts,” Rapid Communications in Mass Spectrometry 18, no. 1 (2004): 49–58, 10.1002/rcm.1276. 14689559

[62] S. Guan , M. F. Moran , and B. Ma , “Prediction of LC‐MS/MS Properties of Peptides From Sequence by Deep Learning,” Molecular & Cellular Proteomics 18, no. 10 (2019): 2099–2107, 10.1074/mcp.TIR119.001412. 31249099 PMC6773555

[63] F. Vilenne , A. Agten , S. Appeltans , G. Ertaylan , and D. Valkenborg , “CPred: Charge State Prediction for Modified and Unmodified Peptides in Electrospray Ionization,” Analytical Chemistry 96, no. 36 (2024): 14382–14392, 10.1021/acs.analchem.4c01107. 39189425

[64] J. M. Hogan , R. Higdon , N. Kolker , and E. Kolker , “Charge State Estimation for Tandem Mass Spectrometry Proteomics,” Omics: a Journal of Integrative Biology 9, no. 3 (2005): 233–250, 10.1089/omi.2005.9.233. 16209638

[65] A. A. Klammer , C. C. Wu , M. J. MacCoss , and W. S. Noble , “Peptide Charge State Determination for Low‐Resolution Tandem Mass Spectra,” in 2005 IEEE Computational Systems Bioinformatics Conference (CSB'05) (2005), 175–185, 10.1109/CSB.2005.44. 16447975

[66] R. G. Sadygov , Z. Hao , and A. F. R. Huhmer , “Charger: Combination of Signal Processing and Statistical Learning Algorithms for Precursor Charge‐State Determination From Electron‐Transfer Dissociation Spectra,” Analytical Chemistry 80, no. 2 (2008): 376–386, 10.1021/ac071332q. 18081262

[67] P. C. Carvalho , D. Cociorva , C. C. L. Wong , M. D. G. D. C. Carvalho , V. C. Barbosa , and J. R. Yates , “Charge Prediction Machine: Tool for Inferring Precursor Charge States of Electron Transfer Dissociation Tandem Mass Spectra,” Analytical Chemistry 81, no. 5 (2009): 1996–2003, 10.1021/ac8025288. 19203245 PMC2865572

[68] V. Sharma , J. K. Eng , S. Feldman , P. D. Von Haller , M. J. Maccoss , and W. S. Noble , “Precursor Charge State Prediction for Electron Transfer Dissociation Tandem Mass Spectra,” Journal of Proteome Research 9, no. 10 (2010): 5438–5444, 10.1021/pr1006685. 20731383 PMC2966942

[69] H. Liu , J. Zhang , H. Sun , C. Xu , Y. Zhu , and H. Xie , “The Prediction of Peptide Charge States for Electrospray Ionization in Mass Spectrometry,” Procedia Environmental Sciences 8 (2011): 483–491, 10.1016/j.proenv.2011.10.076.

[70] F. Meier , N. D. Köhler , A.‐D. Brunner , et al., “Deep Learning the Collisional Cross Sections of the Peptide Universe from a Million Experimental Values,” Nature Communications 12, no. 1 (2021): 1185, 10.1038/s41467-021-21352-8. PMC7896072 33608539

[71] M. Chen , P. Zhu , Q. Wan , et al., “High‐Coverage Four‐Dimensional Data‐Independent Acquisition Proteomics and Phosphoproteomics Enabled by Deep Learning‐Driven Multidimensional Predictions,” Analytical Chemistry 95, no. 19 (2023): 7495–7502, 10.1021/acs.analchem.2c05414. 37126374

[72] AlphaPeptDeep development team . “AlphaPeptDeep Charge Model,” *GitHub* (2024), https://github.com/MannLabs/alphapeptdeep/blob/main/peptdeep/model/charge.py.

[73] S. Siami‐Namini , N. Tavakoli , and A. S. Namin , “The Performance of LSTM and BiLSTM in Forecasting Time Series,” in 2019 IEEE International Conference on Big Data (Big Data) (2019), 3285–3292, 10.1109/BigData47090.2019.9005997.

[74] M. R. Rezaei‐Dastjerdehei , A. Mijani , and E. Fatemizadeh , “Addressing Imbalance in Multi‐Label Classification Using Weighted Cross Entropy Loss Function,” in 2020 27th National and 5th International Iranian Conference on Biomedical Engineering (ICBME) (2020), 333–338, 10.1109/ICBME51989.2020.9319440.

[75] D. P. Zolg , M. Wilhelm , T. Schmidt , et al., “ProteomeTools: Systematic Characterization of 21 Post‐Translational Protein Modifications by Liquid Chromatography Tandem Mass Spectrometry (LC‐MS/MS) Using Synthetic Peptides,” Molecular & Cellular Proteomics 17, no. 9 (2018): 1850–1863, 10.1074/mcp.TIR118.000783. 29848782 PMC6126394

[76] C. Wu , W. F. Siems , J. Klasmeier , and H. H. Hill , “Separation of Isomeric Peptides Using Electrospray Ionization/High‐resolution Ion Mobility Spectrometry,” Analytical Chemistry 72, no. 2 (2000): 391–395, 10.1021/ac990601c. 10658335

[77] K. Y. Kartowikromo , O. E. Olajide , and A. M. Hamid , “Collision Cross Section Measurement and Prediction Methods in Omics,” Journal of Mass Spectrometry 58, no. 9 (2023): e4973, 10.1002/jms.4973. 37620034 PMC10530098

[78] F. Meier , M. A. Park , and M. Mann , “Trapped Ion Mobility Spectrometry and Parallel Accumulation–Serial Fragmentation in Proteomics,” Molecular & Cellular Proteomics 20 (2021):100138, 10.1016/j.mcpro.2021.100138. 34416385 PMC8453224

[79] S. J. Valentine , A. E. Counterman , and D. E. Clemmer , “A Database of 660 Peptide Ion Cross Sections: Use of Intrinsic Size Parameters for Bona Fide Predictions of Cross Sections,” Journal of the American Society for Mass Spectrometry 10, no. 11 (1999): 1188–1211, 10.1016/S1044-0305(99)00079-3. 10536822

[80] B. Wang , S. Valentine , M. Plasencia , S. Raghuraman , and X. Zhang , “Artificial Neural Networks for the Prediction of Peptide Drift Time in Ion Mobility Mass Spectrometry,” BMC Bioinformatics 11 (2010): 1, 10.1186/1471-2105-11-182. PMC2874804 20380738

[81] A. R. Shah , K. Agarwal , E. S. Baker , et al., “Machine Learning Based Prediction for Peptide Drift Times in Ion Mobility Spectrometry,” Bioinformatics 26, no. 13 (2010): 1601–1607, 10.1093/bioinformatics/btq245. 20495001 PMC2913656

[82] Y. Zhang , Q. Jin , S. Wang , and R. Ren , “Modeling and Prediction of Peptide Drift Times in Ion Mobility Spectrometry Using Sequence‐Based and Structure‐Based Approaches,” Computers in Biology and Medicine 41, no. 5 (2011): 272–277, 10.1016/j.compbiomed.2011.03.002. 21439562

[83] J. Wang , Y. Yu , Y. Zhao , D. Zhang , and J. Li , “Evaluation and Integration of Existing Methods for Computational Prediction of Allergens,” *BMC Bioinformatics* 14 (2013): S1, 10.1186/1471-2105-14-S8-S9. PMC3599076 23514097

[84] D. H. Ross , J. H. Cho , and L. Xu , “Breaking Down Structural Diversity for Comprehensive Prediction of Ion‐Neutral Collision Cross Sections,” Analytical Chemistry 92, no. 6 (2020): 4548–4557, 10.1021/acs.analchem.9b05772. 32096630

[85] Y. V. Samukhina , D. D. Matyushin , O. I. Grinevich , and A. K. Buryak , “A Deep Convolutional Neural Network for Prediction of Peptide Collision Cross Sections in Ion Mobility Spectrometry,” Biomolecules 11, no. 12 (2021): 1904, 10.3390/biom11121904. 34944547 PMC8699202

[86] D. Teschner , D. Gomez‐Zepeda , A. Declercq , et al., “Ionmob: A Python Package for Prediction of Peptide Collisional Cross‐Section Values,” Bioinformatics 39, no. 9 (2023): btad486, 10.1093/bioinformatics/btad486. 37540201 PMC10521631

[87] G. He , Q. He , J. Cheng , R. Yu , J. Shuai , and Y. Cao , “ProPept‐MT: A Multi‐Task Learning Model for Peptide Feature Prediction,” International Journal of Molecular Sciences 25, no. 13 (2024): 7237, 10.3390/ijms25137237. 39000344 PMC11241495

[88] J. McKetney , I. J. Miller , A. Hutton , P. Sinitcyn , J. J. Coon , and J. G. Meyer , “Deep Learning Predicts Non‐Normal Peptide FAIMS Mobility Distributions Directly from Sequence,” BioRxiv (2024): 2024.09.11.612538, 10.1101/2024.09.11.612538. PMC11800176 39865577

[89] R. Fu , Z. Zhang , and L. Li , “Using LSTM and GRU Neural Network Methods for Traffic Flow Prediction,” in 2016 31st Youth Academic Annual Conference of Chinese Association of Automation (YAC) (2016), 324–328, 10.1109/YAC.2016.7804912.

[90] D. Rathore , F. Aboufazeli , Y. Huang , V. Kolli , G. S. Fernando , and E. D. Dodds , “Ion Dissociation Methods in Proteomics,” Encyclopedia of Analytical Chemistry: Applications, Theory and Instrumentation (2015): 1–26, 10.1002/9780470027318.a9310.

[91] M. B. Hamaneh , A. Y. Ogurtsov , O. I. Obolensky , and Y.i‐K. Yu , “Systematic Assessment of Deep Learning‐Based Predictors of Fragmentation Intensity Profiles,” Journal Proteome Research 23, no. 6 (2024): 1983–1999, 10.1021/acs.jproteome.3c00857. PMC11165591 38728051

[92] K. Liu , S. Li , L. Wang , Y. Ye , and H. Tang , “Full‐Spectrum Prediction of Peptides Tandem Mass Spectra Using Deep Neural Network,” Analytical Chemistry 92, no. 6 (2020): 4275–4283, 10.1021/acs.analchem.9b04867. 32053352 PMC8057055

[93] N. P. Dong , Y.i‐Z. Liang , Q. S. Xu , et al., “Prediction of Peptide Fragment Ion Mass Spectra by Data Mining Techniques,” Analytical Chemistry 86, no. 15 (2014): 7446–7454, 10.1021/ac501094m. 25032905

[94] C. Tarn and W. F. Zeng , “pDeep3: Toward More Accurate Spectrum Prediction With Fast Few‐Shot Learning,” Analytical Chemistry 93, no. 14 (2021): 5815–5822, 10.1021/acs.analchem.0c05427. 33797898

[95] A. Vaswani , N. Shazeer , N. Parmar , et al., “Attention Is All You Need,” Advances in Neural Information Processing Systems 30 (2017): 1706.03762, https://proceedings.neurips.cc/paper_files/paper/2017/file/3f5ee243547dee91fbd053c1c4a845aa‐Paper.pdf.

[96] 3Blue1Brown, "Attention in Transformers, Visually Explained | DL6," *YouTube* (2024), https://youtu.be/eMlx5fFNoYc?feature=shared.

[97] Y. Liu , E. Jun , Q. Li , and J. Heer , “Latent Space Cartography: Visual Analysis of Vector Space Embeddings,” Computer Graphics Forum 38, no. 3 (2019): 67–78, 10.1111/cgf.13672.

[98] U. H. Toprak , L. C. Gillet , A. Maiolica , P. Navarro , A. Leitner , and R. Aebersold , “Conserved Peptide Fragmentation as a Benchmarking Tool for Mass Spectrometers and a Discriminating Feature for Targeted Proteomics,” Molecular &Cellular Proteomics 13 (2014): 2056–2071, 10.1074/mcpO113.036475. 24623587 PMC4125737

[99] P. Mallick , M. Schirle , S. S. Chen , et al., “Computational Prediction of Proteotypic Peptides for Quantitative Proteomics,” Nature Biotechnology 25, no. 1 (2007): 125–131, 10.1038/nbt1275. 17195840

[100] V. A. Fusaro , D. R. Mani , J. P. Mesirov , and S. A. Carr , “Prediction of High‐Responding Peptides for Targeted Protein Assays by Mass Spectrometry,” Nature Biotechnology 27, no. 2 (2009): 190–198, 10.1038/nbt.1524. PMC2753399 19169245

[101] H. Cheng , B. Rao , L. Liu , et al., “PepFormer: End‐to‐End Transformer‐Based Siamese Network to Predict and Enhance Peptide Detectability Based on Sequence Only,” Analytical Chemistry 93, no. 16 (2021): 6481–6490, 10.1021/acs.analchem.1c00354. 33843206

[102] J. Yang , Z. Cheng , F. Gong , and Y. Fu , “DeepDetect: Deep Learning of Peptide Detectability Enhanced by Peptide Digestibility and Its Application to DIA Library Reduction,” Analytical Chemistry 95, no. 15 (2023): 6235–6243, 10.1021/acs.analchem.2c03662. 36908083

[103] A. B. Dincer , Y. Lu , D. K. Schweppe , S. Oh , and W. S. Noble , “Reducing Peptide Sequence Bias in Quantitative Mass Spectrometry Data With Machine Learning,” Journal of Proteome Research 21, no. 7 (2022): 1771–1782, 10.1021/acs.jproteome.2c00211. 35696663 PMC9531543

[104] O. Al Musaimi , O. M. M. Valenzo , and D. R. Williams , “Prediction of Peptides Retention Behavior in Reversed‐Phase Liquid Chromatography Based on Their Hydrophobicity,” Journal Separation Science 46, no. 2 (2023): 2200743, 10.1002/jssc.202200743. PMC10098489 36349538

[105] Y. W. Lao , M. Gungormusler‐Yilmaz , S. Shuvo , T. Verbeke , V. Spicer , and O. V. Krokhin , “Chromatographic Behavior of Peptides Containing Oxidized Methionine Residues in Proteomic LC–MS Experiments: Complex Tale of a Simple Modification,” Journal of Proteomics 125 (2015): 131–139, 10.1016/j.jprot.2015.05.018. 26025879

[106] C. Bich , S. Baer , M. C. Jecklin , and R. Zenobi , “Probing the Hydrophobic Effect of Noncovalent Complexes by Mass Spectrometry,” Journal of the American Society for Mass Spectrometry 21, no. 2 (2010): 286–289, 10.1016/j.jasms.2009.10.012. 19931466

[107] S. Amrhein , S. A. Oelmeier , F. Dismer , and J. Hubbuch , “Molecular Dynamics Simulations Approach for the Characterization of Peptides With Respect to Hydrophobicity,” Journal of Physical Chemistry B 118, no. 7 (2014): 1707–1714, 10.1021/jp407390f. 24506060

[108] S. Simm , J. Einloft , O. Mirus , and E. Schleiff , “50 years of Amino Acid Hydrophobicity Scales: Revisiting the Capacity for Peptide Classification,” Biological Research 49, no. 1 (2016): 31, 10.1186/s40659-016-0092-5. 27378087 PMC4932767

[109] J. L. M. Hermens , J. H. M. De Bruijn , and D. N. Brooke , “The Octanol–Water Partition Coefficient: Strengths and Limitations,” Environmental Toxicology and Chemistry 32, no. 4 (2013): 732–733, 10.1002/etc.2141. 23508402

[110] A. Paschke , P. L. Neitzel , W. Walther , and G. Schüürmann , “Octanol/Water Partition Coefficient of Selected Herbicides: Determination Using Shake‐Flask Method and Reversed‐Phase High‐Performance Liquid Chromatography,” Journal of Chemical & Engineering Data 49, no. 6 (2004): 1639–1642, 10.1021/je049947x.

[111] C. Isert , J. C. Kromann , N. Stiefl , G. Schneider , and R. A. Lewis , “Machine Learning for Fast, Quantum Mechanics‐Based Approximation of Drug Lipophilicity,” ACS Omega 8 (2023): 2046–2056, 10.1021/acsomega.2c05607. 36687099 PMC9850743

[112] M. Isik , T. D. Bergazin , T. Fox , A. Rizzi , J. D. Chodera , and D. L. Mobley , “Assessing the Accuracy of Octanol–Water Partition Coefficient Predictions in the SAMPL6 Part II Log P Challenge,” Journal of Computer‐Aided Molecular Design 34, no. 4 (2020): 335–370, 10.1007/s10822-020-00295-0. 32107702 PMC7138020

[113] C. N. Manners , D. W. Payling , and D. A. Smith , “Distribution Coefficient, a Convenient Term for the Relation of Predictable Physico‐Chemical Properties to Metabolic Processes,” Xenobiotica 18, no. 3 (1988): 331–350, 10.3109/00498258809041669. 3289270

[114] N. Gulyaeva , A. Zaslavsky , P. Lechner , A. Chait , and B. Zaslavsky , “pH Dependence of the Relative Hydrophobicity and Lipophilicity of Amino Acids and Peptides Measured by Aqueous Two‐Phase and Octanol–Buffer Partitioning,” Journal of Peptide Research 61, no. 2 (2003): 71–79, 10.1034/j.1399-3011.2003.00037.x. 12492901

[115] I. V. Tetko and P. Bruneau , “Application of ALOGPS to Predict 1‐Octanol/Water Distribution Coefficients, logP, and logD, of AstraZeneca in‐House Database,” Journal of Pharmaceutical Sciences 93, no. 12 (2004): 3103–3110, 10.1002/jps.20217. 15514985

[116] I. V. Tetko and G. I. Poda , “Application of ALOGPS 2.1 to Predict Log D Distribution Coefficient for Pfizer Proprietary Compounds,” Journal of Medicinal Chemistry 47, no. 23 (2004): 5601–5604, 10.1021/jm049509l. 15509156

[117] I. V. Tetko , V. Y. Tanchuk , T. N. Kasheva , and A. E. P. Villa , “Estimation of Aqueous Solubility of Chemical Compounds Using E‐State Indices,” Journal of Chemical Information and Computer Sciences 41, no. 6 (2001): 1488–1493, 10.1021/ci000392t. 11749573

[118] I. V. Tetko , V. Y. Tanchuk , and A. E. P. Villa , “Prediction of n‐Octanol/Water Partition Coefficients From PHYSPROP Database Using Artificial Neural Networks and E‐State Indices,” Journal of Chemical Information and Computer Sciences 41, no. 5 (2001): 1407–1421, 10.1021/ci010368v. 11604042

[119] I. V. Tetko and V. Y. Tanchuk , “Application of Associative Neural Networks for Prediction of Lipophilicity in ALOGPS 2.1 Program,” Journal of Chemical Information and Computer Sciences 42, no. 5 (2002): 1136–1145, 10.1021/ci025515j. 12377001

[120] V. Erckes and C. Steuer , “A Story of Peptides, Lipophilicity and Chromatography—Back and Forth in Time,” RSC Medicinal Chemistry 13, no. 6 (2022): 676–687, 10.1039/d2md00027j. 35800203 PMC9215158

[121] J.‐A. Fuchs , F. Grisoni , M. Kossenjans , J. A. Hiss , and G. Schneider , “Lipophilicity Prediction of Peptides and Peptide Derivatives by Consensus Machine Learning,” Medicinal Chemistry Communications 9, no. 9 (2018): 1538–1546, 10.1039/C8MD00370J. 30288227 PMC6151477

[122] A. Visconti , G. Ermondi , G. Caron , and R. Esposito , “Prediction and Interpretation of the Lipophilicity of Small Peptides,” Journal of Computer‐Aided Molecular Design 29, no. 4 (2015): 361–370, 10.1007/s10822-015-9829-4. 25577035

[123] S. Lobo , “Is There Enough Focus on Lipophilicity in Drug Discovery?,” Expert Opinion on Drug Discovery 15, no. 3 (2020): 261–263, 10.1080/17460441.2020.1691995. 31736369

[124] T. T. V. Tran , H. Tayara , and K. T. Chong , “Recent Studies of Artificial Intelligence on in Silico Drug Absorption,” Journal of Chemical Information and Modeling 63, no. 20 (2023): 6198–6211, 10.1021/acs.jcim.3c00960. 37819031

[125] N. Ulrich , K.‐U. Goss , and A. Ebert , “Exploring the Octanol–Water Partition Coefficient Dataset Using Deep Learning Techniques and Data Augmentation,” Communications Chemistry 4, no. 1 (2021): 90, 10.1038/s42004-021-00528-9. PMC9814212 36697535

[126] J. Kraml , A. S. Kamenik , F. Waibl , M. Schauperl , and K. R. Liedl , “Solvation Free Energy as a Measure of Hydrophobicity: Application to Serine Protease Binding Interfaces,” Journal of Chemical Theory and Computation 15, no. 11 (2019): 5872–5882, 10.1021/acs.jctc.9b00742. 31589427 PMC7032847

[127] A. Mahajan , A. S. Rawat , N. Bhatt , and M. K. Chauhan , “Structural Modification of Proteins and Peptides,” Indian Journal of Pharmaceutical Education and Research 48, no. 3 (2014): 34–47, 10.5530/ijper.48.3.6.

[128] E. F. Mcdonald , T. Jones , L. Plate , J. Meiler , and A. Gulsevin , “Benchmarking AlphaFold2 on Peptide Structure Prediction,” Structure (London, England) 31, no. 1 (2023): 111–119.e2, 10.1016/j.str.2022.11.012. PMC9883802 36525975

[129] J. Jumper , R. Evans , A. Pritzel , et al., “Highly Accurate Protein Structure Prediction With AlphaFold,” Nature 596, no. 7873 (2021): 583–589, 10.1038/s41586-021-03819-2. 34265844 PMC8371605

[130] J. Pereira , A. J. Simpkin , M. D. Hartmann , D. J. Rigden , R. M. Keegan , and A. N. Lupas , “High‐Accuracy Protein Structure Prediction in CASP14,” Proteins 89, no. 12 (2021): 1687–1699, 10.1002/prot.26171. 34218458

[131] I. Sample, “DeepMind AI Cracks 50‐Year‐Old Problem of Protein Folding,” The Guardian (2020), https://www.theguardian.com/technology/2020/nov/30/deepmind‐ai‐cracks‐50‐year‐old‐problem‐of‐biology‐research.

[132] H. Briggs, "One of Biology's Biggest Mysteries ‘largely Solved’," *BBC* *News* (2020), https://www.bbc.com/news/science‐environment‐55133972.

[133] A. Lamiable , P. Thévenet , J. Rey , M. Vavrusa , P. Derreumaux , and P. Tufféry , “PEP‐FOLD3: Faster De Novo Structure Prediction for Linear Peptides in Solution and in Complex,” Nucleic Acids Research 44, no. W1 (2016): W449–W454, 10.1093/nar/gkw329. 27131374 PMC4987898

[134] R. Wu , F. Ding , R. Wang , et al., High‐Resolution De Novo Structure Prediction From Primary Sequence," *BioRxiv* (2022): 2022.07.21.500999, 10.1101/2022.07.21.500999.

[135] M. Baek , F. Dimaio , I. Anishchenko , et al., “Accurate Prediction of Protein Structures and Interactions Using a Three‐Track Neural Network,” Science 373, no. 6557 (2021): 871–876, 10.1126/science.abj8754. 34282049 PMC7612213

[136] P. B. Timmons and C. M. Hewage , “APPTEST Is a Novel Protocol for the Automatic Prediction of Peptide Tertiary Structures,” Briefings in Bioinformatics 22, no. 6 (2021): bbab308, 10.1093/bib/bbab308. 34396417 PMC8575040

[137] J. Abramson , J. Adler , J. Dunger , et al., “Accurate Structure Prediction of Biomolecular Interactions With AlphaFold 3,” Nature 630, no. 8016 (2024): 493–500, 10.1038/s41586-024-07487-w. 38718835 PMC11168924

[138] R. Krishna , J. Wang , W. Ahern , et al., “Generalized Biomolecular Modeling and Design With RoseTTAFold all‐Atom,” Science 384, no. 6693 (2024): eadl2528, 10.1126/science.adl2528.38452047

[139] F.‐A. Croitoru , V. Hondru , R. T. Ionescu , and M. Shah , “Diffusion Models in Vision: A Survey,” IEEE Transactions on Pattern Analysis and Machine Intelligence 45, no. 9 (2023): 10850–10869, 10.1109/TPAMI.2023.3261988. 37030794

[140] C. Discovery , J. Boitreaud , J. Dent, et al., "Chai‐1: Decoding the Molecular Interactions of Life," *BioRxiv* (2024): 2024.10.10.615955,, 10.1101/2024.10.10.615955.

[141] datacamp, "datacamp.com," (2024), https://www.datacamp.com/.

[142] freeCodeCamp, freeCodeCamp," (2024), https://www.freecodecamp.org/.

[143] learnpython, "learnpython.org," (2024), https://www.learnpython.org/.

[144] ONEIROS, Keras," (2024), https://keras.io/.

[145] PyTorch Foundation, “PyTorch,” (2024), https://pytorch.org/.

[146] M. Abadi , A. Agarwal , P. Barham , et al., "TensorFlow: Large‐Scale Machine Learning on Heterogeneous Systems," *arXiv preprint* (2016): 1603.04467, 10.48550/arXiv.1603.04467.

[147] HuggingFace, "HuggingFace .co," (2024), https://huggingface.co/.

[148] W. Fondrie , W. N. A. Bittremieux , J. A. Sanders , M. Yilmaz , and A. B. Dincer , “depthcharge,” *GitHub* (2024), https://github.com/wfondrie/depthcharge.

[149] A. Declercq , R. Bouwmeester , C. Chiva , et al., “Updated MS^2^PIP Web Server Supports Cutting‐Edge Proteomics Applications,” Nucleic Acids Research 51, no. W1 (2023): W338–W342, 10.1093/nar/gkad335. 37140039 PMC10320101

[150] Weights & Biases, “Weights & Biases,” (2024), https://wandb.ai/site/.

[151] T. G. Rehfeldt , R. Gabriels , R. Bouwmeester , et al., “ProteomicsML: An Online Platform for Community‐Curated Data Sets and Tutorials for Machine Learning in Proteomics,” Journal of Proteome Research 22, no. 2 (2023): 632–636, 10.1021/acs.jproteome.2c00629. 36693629 PMC9903315

[152] O. Shouman , W. Gabriel , V. Giurcoiu , T. Schmidt , and N. Khalek , "DLOmix," *GitHub* (2024), https://github.com/wilhelm‐lab/dlomix.

[153] M. Krenn , Q. Ai , S. Barthel , et al., “SELFIES and the Future of Molecular String Representations,” Patterns 3, no. 10 (2022): 100588, 10.1016/j.patter.2022.100588. 36277819 PMC9583042

[154] Z. Liu , J. Wang , Y. Luo , S. Zhao , W. Li , and S. Z. Li , “Efficient Prediction of Peptide Self‐Assembly Through Sequential and Graphical Encoding,” Briefings in Bioinformatics 24, no. 6 (2023): bbad409, 10.1093/bib/bbad409. 37974507

[155] A. Kensert , R. Bouwmeester , K. Efthymiadis , P. Van Broeck , G. Desmet , and D. Cabooter , “Graph Convolutional Networks for Improved Prediction and Interpretability of Chromatographic Retention Data,” Analytical Chemistry 93, no. 47 (2021): 15633–15641, 10.1021/acs.analchem.1c02988. 34780168

[156] H. Xu , J. Lin , D. Zhang , and F. Mo , “Retention Time Prediction for Chromatographic Enantioseparation by Quantile Geometry‐Enhanced Graph Neural Network,” Nature Communications 14, no. 1 (2023): 3095, 10.1038/s41467-023-38853-3. PMC10227049 37248214

[157] P. Lecca and M. Lecca , “Graph Embedding and Geometric Deep Learning Relevance to Network Biology and Structural Chemistry,” Frontiers in Artificial Intelligence 6 (2023): 1256352, 10.3389/frai.2023.1256352. PMC10687447 38035201

[158] M. Ekvall , P. Truong , W. Gabriel , M. Wilhelm , and L. Käll , “Prosit Transformer: A Transformer for Prediction of MS2 Spectrum Intensities,” Journal of Proteome Research 21, no. 5 (2022): 1359–1364, 10.1021/acs.jproteome.1c00870. 35413196 PMC9087333

[159] C. Guntuboina , A. Das , P. Mollaei , S. Kim , and A. Barati Farimani , “Peptidebert: A Language Model Based on Transformers for Peptide Property Prediction,” Journal of Physical Chemistry Letters 14, no. 46 (2023): 10427–10434, 10.1021/acs.jpclett.3c02398. 37956397 PMC10683064

[160] N. Brandes , D. Ofer , Y. Peleg , N. Rappoport , and M. Linial , “ProteinBERT: A Universal Deep‐Learning Model of Protein Sequence and Function,” Bioinformatics 38, no. 8 (2022): 2102–2110, 10.1093/bioinformatics/btac020. 35020807 PMC9386727

[161] J. Yang , K. Zhou , Y. Li , and Z. Liu , “Generalized Out‐of‐Distribution Detection: A Survey,” *International Journal of Computer Vision* 132.12 (2024): 5635‐5662, 10.1007/s11263-024-02117-4.

[162] A. N. Angelopoulos and S. Bates , “A Gentle Introduction to Conformal Prediction and Distribution‐Free Uncertainty Quantification,” arXiv preprint (2021), 2107.07511, 10.48550/arXiv.2107.07511.

[163] N. Abdul‐Khalek , R. Wimmer , M. T. Overgaard , and S. G. Echers , “Decoding the Impact of Neighboring Amino Acid on MS Intensity Output Through Deep Learning,” Journal of Proteomics 309 (2024): 105322, 10.1016/j.jprot.2024.105322. 39341565

[164] S. Tiwary , R. Levy , P. Gutenbrunner , et al., “High‐Quality MS/MS Spectrum Prediction for Data‐Dependent and Data‐Independent Acquisition Data Analysis,” Nature Methods 16, no. 6 (2019): 519–525, 10.1038/s41592-019-0427-6. 31133761

[165] Q. Dickinson and J. G. Meyer , “Positional SHAP (PoSHAP) for Interpretation of Machine Learning Models Trained From Biological Sequences,” PLoS Computational Biology 18, no. 1 (2022): e1009736, 10.1371/journal.pcbi.1009736. 35089914 PMC8797255

[166] K. Lee , D. Ippolito , A. Nystrom , et al., “Deduplicating Training Data Makes Language Models Better,” in Proceedings of the 60th Annual Meeting of the Association for Computational Linguistics (Vol. 1: Long Papers), ed. S. Muresan , P. Nakov , and A. Villavicencio , et al. (Association for Computational Linguistics, 2022), 8424–8445, 10.18653/v1/2022.acl-long.577.

[167] O. Shouman , W. Gabriel , V. G. Giurcoiu , V. Sternlicht , and M. Wilhelm , “PROSPECT: Labeled Tandem Mass Spectrometry Dataset for Machine Learning in Proteomics,” Advances in Neural Information Processing Systems 35 (2022): 32882–32896, https://proceedings.neurips.cc/paper_files/paper/2022/file/d42db1f74df54cb992b3956eb7f15a6f‐Paper‐Datasets_and_Benchmarks.pdf.

[168] W. Gabriel , O. Shouman , E. A. Schröder , F. Bö , and M. Wilhelm , “PROSPECT PTMs: Rich Labeled Tandem Mass Spectrometry Dataset of Modified Peptides for Machine Learning in Proteomics,” Advances in Neural Information Processing Systems 37 (2024): 131154–131196, https://proceedings.neurips.cc/paper_files/paper/2022/file/d42db1f74df54cb992b3956eb7f15a6f‐Paper‐Datasets_and_Benchmarks.pdf.

[169] J. Zhou , S. Chen , J. Xia , et al., “NovoBench: Benchmarking Deep Learning‐based De Novo Peptide Sequencing Methods in Proteomics,” *arXiv preprint (2024): 2406.11906*, 10.48550/arXiv.2406.11906.

[170] L. Lautenbacher , K. L. Yang , T. Kockmann , et al., “Koina: Democratizing Machine Learning for Proteomics Research,” *BioRxiv* (2024): 2024.06.01.596953, , 10.1101/2024.06.01.596953. PMC12606132 41219230

