# Supplementary material for: Peptide Property Prediction for Mass Spectrometry Using AI: An Introduction to State of the Art Models
Source: Proteomics. 2025 Apr 10;25(9-10):e202400398. doi: 10.1002/pmic.202400398 (PMC12076536; doi:10.1002/pmic.202400398)
Supplement: Supplementary file 1 — Supporting Information [file PMIC-25-e202400398-s001.docx]

**Peptide Property Prediction in Mass Spectrometry Using AI:**

An Introduction to state of the art models

**Jesse Angelis^1^, Eva Ayla Schröder^1^, Zixuan Xiao^1^, Wassim Gabriel ^1^ and Mathias Wilhelm^1,2*^**

*^1^Computational Mass Spectrometry, Technical University of Munich, Freising, 85354, Germany*

*^2^Munich Data Science Institute (MDSI), Technical University of Munich, Garching,*

*85748, Germany*

**Sporting Information**

*Corresponding author

Prof. Dr. Mathias Wilhelm,

Maximus-von-Imhof-Forum 3

D - 85354 Freising mathias.wilhelm@tum.de

[Types of Machine Learning (ML) 3](#_Toc190858666)

[Supervised learning 3](#_Toc190858667)

[Unsupervised learning 3](#_Toc190858668)

[Reinforcement learning 3](#_Toc190858669)

[Feature engineering 3](#_Toc190858670)

[Deep Learning (DL) 3](#_Toc190858671)

[Explainable AI (XAI) 4](#_Toc190858672)

[Training Data Requirements 5](#_Toc190858673)

[The Loss Function 6](#_Toc190858674)

[Training Process 7](#_Toc190858675)

[Embeddings 8](#_Toc190858676)

[Metrics 8](#_Toc190858677)

[Classification metrics 9](#_Toc190858678)

[Accuracy 9](#_Toc190858679)

[Area Under the receiver operating characteristic (ROC) Curve (AUC) 9](#_Toc190858680)

[F1 Score 10](#_Toc190858681)

[Mathews correlation coefficient (MCC) 11](#_Toc190858682)

[Regression metrics 12](#_Toc190858683)

[Mean absolute error (MAE) 12](#_Toc190858684)

[Mean squared error (MSE) 12](#_Toc190858685)

[Pearson correlation coefficient (PCC) 12](#_Toc190858686)

[Median absolute percent error (MAPE) 12](#_Toc190858687)

# **Types of Machine Learning (ML)**

## **Supervised learning**

Supervised learning uses labeled data, where known input-output pairs are provided to train the model. By optimizing a function applied to the input, it can predict outcomes for new data. [[1]](https://www.zotero.org/google-docs/?RrPaVx) This is particularly relevant in mass spectrometry (MS) for predicting peptide properties, where labeled training data is often available.

## **Unsupervised learning**

Unsupervised learning works with unlabeled data and aims to uncover hidden patterns, which can be useful for detecting clusters in peptide datasets. Due to the missing labels these algorithms lack the error estimates supervised learners use for optimization. [[2]](https://www.zotero.org/google-docs/?F5jKF2)

## **Reinforcement learning**

Reinforcement learning is an approach that receives feedback from its environment. Through trial-and-error it optimizes its underlying function by maximizing a reward function. [[3]](https://www.zotero.org/google-docs/?vnXD5X) While less common in proteomics, it has potential for optimizing experimental procedures or complex peptide behavior.

# **Feature engineering**

Feature engineering is the selection of the most relevant variables (features) from data. In traditional ML, this is often done explicitly by domain experts, allowing for better explainability of model decisions. [[4]](https://www.zotero.org/google-docs/?HZ9cjG) This explainability is an advantage, because understanding the contribution of each feature increases confidence in the model, as they can be checked for their theoretic physico-chemical soundness. [[5]](https://www.zotero.org/google-docs/?FQBJxL) While trial and error can help to reduce a feature set to the most relevant ones, this approach is limited by our understanding of the underlying mechanisms.

# **Deep Learning (DL)**

Deep learning (DL), a subset of machine learning, utilizes artificial neural networks inspired by the structure of neurons in the human brain to automatically learn high-dimensional data representations. [[6–8]](https://www.zotero.org/google-docs/?AwzS5l) It excels in handling complex input-output mappings and is particularly powerful in reducing data complexity while maintaining relevant information. [[7,9]](https://www.zotero.org/google-docs/?Yw8NaT) One of the simplest DL architectures is the **multilayer perceptron (MLP)**, which uses multiple layers of so-called nonlinear processing units, which work similar to biological neurons. They take signal inputs (in DL: numerical values vs. in biology: chemical stimuli) and calculate an output (in DL: weighted summation vs in biology: adjustment of the membrane potential based on excitatory or inhibitory signals). Each layer takes the output of the previous one as input, allowing the network to learn and represent data at increasingly abstract levels. [[9]](https://www.zotero.org/google-docs/?2FcngP) This enables DL models to discover more abstract features at higher representation levels. [[10]](https://www.zotero.org/google-docs/?NmJj9j) Its ability to recognize hidden patterns in large datasets has made it a disruptive technology in artificial intelligence. [[8]](https://www.zotero.org/google-docs/?HmWWFf)

In MS-based proteomics, DL models such as long short-term memory (LSTM) networks [[11]](https://www.zotero.org/google-docs/?LCDnPn), have been applied to predict various peptide properties, including retention times, charge state distributions, and fragment ion intensities in mass spectrometry. [[12]](https://www.zotero.org/google-docs/?rWy3y6) LSTMs, which are well-suited for sequential data, outperform traditional methods in both accuracy and efficiency. [[13,14]](https://www.zotero.org/google-docs/?WUlSWg)

A key advantage of DL is its ability to perform **implicit feature engineering,** automatically extracting relevant features from raw data without requiring manual intervention. This is particularly useful in proteomics, where high-dimensional MS data often requires extensive preprocessing and domain-specific feature selection. [[15]](https://www.zotero.org/google-docs/?LBsnIo) By bypassing the need for explicit feature engineering, DL models reduce the burden on researchers and allow for the discovery of previously hidden patterns within complex datasets.

# **Explainable AI (XAI)**

Making these patterns interpretable for humans is its own field of research, which is commonly referred to as explainable AI (XAI). For instance, in biomedicine XAI has been used to score the importance of physico-chemical properties of signal peptides when predicting secretion efficiencies, allowing for a more informed evaluation of signal peptides. [[16]](https://www.zotero.org/google-docs/?tLppes) Investigating feature importance has also shed light on peptide motifs involved in kinase-substrate interactions. [[17]](https://www.zotero.org/google-docs/?QhjOR7) Additionally, XAI was applied to interpret predictions of peptide retention time in reversed-phase HPLC. It was shown that the most important features were related to the peptides' physico-chemical properties, specifically cross-collisional sections (CCS) and variations in accessible surface area (ASA), both contributing to the macro-coefficient of hydrophobicity. [[18]](https://www.zotero.org/google-docs/?OKyY1u)

In traditional machine learning (ML), explicit feature engineering inherently determines the important features ahead of time. DL models, despite their high accuracy, often struggle with interpretability, turning them into "black boxes". This challenge has spurred active research aimed at developing methods to make DL models more explainable. [[15,19,20]](https://www.zotero.org/google-docs/?VrbYAY)

# **Training Data Requirements**

Traditional ML and DL models have different data requirements, with DL generally considered more "data-hungry" due to its need for larger datasets to perform effectively. [[21,22]](https://www.zotero.org/google-docs/?WkOFGU) This poses a challenge when working with clinical data, which is often difficult to obtain, especially when attempting to represent rare conditions or diseases. [[23]](https://www.zotero.org/google-docs/?xcuTok) In MS applications, the problem of data scarcity is compounded by the complexity of the data itself and the potential imbalance in datasets. Ensuring that the data is balanced, meaning it contains a similar number of positive and negative examples, is crucial to prevent biased predictions and improve model performance. [[17,23]](https://www.zotero.org/google-docs/?UFjtt7)

It is important for a model's prediction task to align with the distribution of the data it was trained on. For instance, training a model on only male data may lead to unreliable results if the model is later used for predictions based on female data. The lack of exposure to female data during training could cause it to make inaccurate or biased predictions. [[24]](https://www.zotero.org/google-docs/?pSLI9I)

A major challenge in ML for MS data is the presence of noise. Noise in MS datasets can arise from various sources, including experimental variations, labeling errors, or the inherent complexity of the biological systems being studied. This noise can negatively impact model accuracy and interpretability, as studies have shown that noisy data, including outliers and mislabeled instances, increases model complexity and reduces performance. [[25–27]](https://www.zotero.org/google-docs/?iAKLEB) In general, ML models aim to explain the variance in the data, much of which is often due to actual underlying properties. However, finer details can be distorted by the inherent randomness of the systems being studied. As the model improves its ability to make predictions based on these properties, it increasingly focuses on random variance. This can, in turn, affect its overall prediction accuracy. If a model learns to recognize patterns that stem from random noise in the training data, it may perform exceptionally well on that data but struggle with new data, as those patterns are not generalizable. This issue is known as **overfitting**. [[28]](https://www.zotero.org/google-docs/?AXvA2L)

To assess the actual predictive capability of a model, datasets are typically split into **training** and **test sets**. The model is optimized on the training set and then evaluated on the test set. It is crucial that both sets accurately represent the problem domain to ensure a meaningful evaluation. However, even if a model performs well on the test set, it does not necessarily guarantee robust performance in real-world scenarios. One common issue that arises is **data leakage**, where non-generalizable information from the test set "leaks" into the model during training, leading to artificially inflated performance metrics. [[29]](https://www.zotero.org/google-docs/?FmiBaO)

In addition to noise and leakage, data quality encompasses many dimensions. According to Black and van Nederpelt, there are as many as 65 factors influencing data quality, further highlighting the complexity of preparing high-quality datasets for machine learning applications. [[30]](https://www.zotero.org/google-docs/?2159QO) Addressing these data issues is crucial for developing reliable models.

# **The Loss Function**

Loss functions quantify the difference between the predicted and actual outcomes. This information is then used to adjust the weights of the model. Thus, loss functions play a crucial role in guiding the learning process. [[31]](https://www.zotero.org/google-docs/?6quTXV) As previously mentioned, noisy labels in training data pose significant challenges for ML models, potentially reducing their generalizability. [[32]](https://www.zotero.org/google-docs/?Wu5V19) Various techniques have been proposed to mitigate these effects, including sample weighting, sample selection, and the use of robust loss functions. [[32]](https://www.zotero.org/google-docs/?z37ynw)

Different types of loss functions, such as generalized cross-entropy [[33]](https://www.zotero.org/google-docs/?X4cush), active passive [[34]](https://www.zotero.org/google-docs/?6lMteg), and asymmetric loss functions [[35]](https://www.zotero.org/google-docs/?v0XrCT), are designed to handle specific types of noise and outliers. This adaptability allows models to learn more effectively from imperfect data. For a general overview of classical loss functions refer to Wang et al. [[31]](https://www.zotero.org/google-docs/?VOK1xe). The choice of loss function can significantly impact both the convergence speed and overall performance of the model, making it essential to select one that aligns with the dataset's characteristics and the specific problem at hand. [[36]](https://www.zotero.org/google-docs/?0SC3xF)

Often, reviews focus solely on model architectures, but understanding loss functions can provide valuable insights into their behavior and optimization. A recent preprint [[37]](https://www.zotero.org/google-docs/?7fRgIG) highlights the critical role of optimizing loss functions in predictive models. Similarly, the *AlphaFold* models [[38,39]](https://www.zotero.org/google-docs/?keRrtj) which were recently recognized by the Nobel Prize in Chemistry, incorporate sophisticated loss functions.

To understand why different loss functions are preferred in certain scenarios, it is crucial to introduce the concept of gradient descent. Supervised ML models derive their predictions through a mathematical equation whose parameters can be iteratively adjusted to achieve the best possible outcome. Each iteration generates a prediction that can be compared to the actual target value, allowing for the calculation of a loss. This loss quantifies the difference between predicted and actual values, guiding the necessary adjustments to the model's parameters. By computing the gradient of the loss, the direction and magnitude of these adjustments can be determined, ensuring that the model converges toward an optimal solution. [[40]](https://www.zotero.org/google-docs/?TU86jm) To gain a more visual and intuitive understanding of the deep learning process, which is beyond the scope of this review, we recommend watching Chapters 1–4 of the Deep Learning playlist by 3Blue1Brown [[41]](https://www.zotero.org/google-docs/?ZELi5s). Even though these YouTube videos are not peer-reviewed, we believe that they are highly valuable resources for any beginner in this field.

# **Training Process**

When developing a machine learning (ML) model, various training methods can be employed based on the task at hand and the available data. A model can either be trained from scratch, starting from an untrained state, or it can leverage knowledge from a pre-trained model through transfer learning. Both approaches have specific uses and advantages.

Training a model **from scratch** means initializing parameters randomly and gradually teaching the model to recognize patterns in the data. This approach is particularly effective for tasks where a large, high-quality dataset is available and when the problem is highly specific. One of the main advantages of training from scratch is the complete flexibility it offers in designing the model architecture, allowing to tailor them to the specific task. However, this method often requires extensive data and computational resources. Additionally, if the dataset is not sufficiently large, there is a higher risk of overfitting. [[42]](https://www.zotero.org/google-docs/?XDpXD3)

**Transfer learning**, in contrast, uses a model that has already been trained on a related task or dataset. Instead of starting with random parameters, the model therefore begins with knowledge that can be adapted to the new problem. This process typically involves selecting a pre-trained model and either using it as a starting point or modifying and retraining specific layers (**fine-tuning**) to fit the new task. Transfer learning is particularly beneficial in proteomics when limited data is available, like for example specific post-translational modifications (PTMs), as it allows the model to leverage existing knowledge to achieve good performance with minimal additional training. However, a main problem is that the pre-trained model may not perfectly align with the new task, limiting the applicability of this approach. [[42,43]](https://www.zotero.org/google-docs/?nKMXGz)

To address the issue of data scarcity, alternative approaches like few-shot and zero-shot learning are being explored. **Few-shot learning** enables models to generalize from a limited number of labeled examples. They make predictions on unseen data by finding key similarities from those examples. Techniques such as embedding learning and meta-learning are often employed to enhance the model's adaptability. [[44]](https://www.zotero.org/google-docs/?hVioyt) On the other hand, **zero-shot** learning takes on the challenge of predicting entirely new classes by leveraging semantic relationships and attributes. This allows models to infer characteristics of unseen categories based on prior knowledge, even without having seen any direct examples. [[45]](https://www.zotero.org/google-docs/?BFVfxc)

# **Embeddings**

To allow calculations to be performed based on amino acid sequences, they need to be represented in a numerical form, which can be done in multiple ways. The simplest and most intuitive method assigns an integer, a so-called **dummy variable**, to each amino acid. However, this creates an implied hierarchy. For instance, if arginine is encoded as 1, cysteine as 2, and leucine as 3, it suggests that cysteine is somehow "between" the other two in terms of properties. When such a hierarchy is arbitrarily chosen and not based on real characteristics, it risks introducing misleading information into the model, which can hinder its learning.

A more effective approach that avoids this issue is **one-hot encoding**. In this method, each amino acid is represented by a binary vector. The result is a two-dimensional matrix where each row corresponds to a position in the peptide sequence, and each column represents a specific amino acid (or vice versa). At each position, the column corresponding to the present amino acid is set to 1, while all other columns remain 0. This approach avoids arbitrary hierarchies and provides a clearer, neutral representation of the sequence. [[46]](https://www.zotero.org/google-docs/?PiZ0GY)

Peptides are defined by their sequential nature forming a kind of sentence where the words are the individual amino acids. This draws similarity to natural language processing. There are multiple ways to embed sentences. One is the so-called **word embedding** where a model is trained to represent a word's meaning as a vector in an n-dimensional space, so that words that are similar have similar vectors. [[47]](https://www.zotero.org/google-docs/?EB2ss3)

**Token embeddings** are similar to word embeddings, however here the word token refers to a meaningful element of a sequence. [[48]](https://www.zotero.org/google-docs/?YMEopy) For example a simplified version of this could be the tokenization of “phosphorylation” as “phosphor-yl-ation” where the word is separated into its meaningful elements “phosphor” refers to phosphorus, “yl” refers to a chemical group, and “ation” refers to the process of adding. In practice these tokens are often a lot smaller only consisting of a few letters. To our knowledge, this type of embedding has not been utilized so far in terms of peptide embedding.

# **Metrics**

To effectively quantify a model's performance, it is crucial to select meaningful metrics. These metrics can serve as both evidence of a model's performance and as loss functions during training. Since most models discussed in this review aim to either classify a given input or perform regression, the following metrics are focused on these two objectives. In classification the performance is usually assessed by the number of true positives (TP), true negatives (TN), false positives (FP), false negatives (FN), or the probability given to a specific class. Regression assessment is usually performed by simply comparing the true vs the predicted values. However, it is important to note that numerous additional metrics exist for both classification and regression tasks, as well as for other types of objectives.

## **Classification metrics**

### **Accuracy**

Accuracy is a straightforward metric used to evaluate the overall correctness of a model by calculating the proportion of correct predictions (both true positives and true negatives) out of the total number of predictions. [[49]](https://www.zotero.org/google-docs/?6NF10X) It is calculated as follows:

| $Accuracy=\frac{TP+TN}{TP+TN+FP+FN}$ | (1) |
| --- | --- |

With $TP$ = True Positive, $TN$ = True Negative, and $FP$ = False Negative Accuracy is a widely used metric for evaluating model performance in classification tasks, often serving as a benchmark to report results, with 1 being the optimal and 0 the worst score. It’s even used as a loss function in some cases. However, accuracy has a notable limitation, particularly when class distributions are imbalanced. This is known as the accuracy paradox. In such cases, a high accuracy score may falsely imply reliable predictions. [[49,50]](https://www.zotero.org/google-docs/?Fv3Bos) For example, consider a dataset with 95% positive and 5% negative samples. A model predicting all instances as positive would achieve an accuracy of 0.95, misleadingly suggesting strong performance. Therefore, relying solely on accuracy as a performance metric, especially with imbalanced data, can lead to misinterpretations, underscoring the need to consider additional metrics. [[49,50]](https://www.zotero.org/google-docs/?JMjDIN)

### **Area Under the receiver operating characteristic (ROC) Curve (AUC)**

AUC measures the ability to distinguish between two classes. In a ROC curve the receiver operating characteristic (true positive rate vs false positive rate) is plotted for every threshold as calculated with:

| $true positive rate=\frac{TP}{TP+FN}$ | (2) |
| --- | --- |
| $false positive rate=\frac{FP}{FP+TN}$ | (3) |

The AUC represents the area beneath this ROC curve, with values ranging from 0 to 1. An ideal model achieves an AUC of 1, indicating perfect classification with only $TP$ and $TN$. An AUC of 0.5 reflects a model that performs no better than random chance. Values below 0.5 suggest that the model's performance is worse than random, indicating a need for significant improvement. [[51,52]](https://www.zotero.org/google-docs/?fxz755)

AUC has several limitations that often make it an unreliable metric for evaluating model performance. For example, it provides an overall summary of performance across all thresholds, including those that are rarely relevant in practical applications, which can mask how well a model performs with applicable thresholds. AUC also disregards the actual predicted values, giving no insight into the model’s actual fit to the data. Thus, models with similar AUC scores can differ significantly in reliability. [[51,52]](https://www.zotero.org/google-docs/?8tFCvS)

### **F1 Score**

The F1 score describes the harmonic mean of precision and recall as calculated with:

| $F1 Score=\frac{2 Precision ・Recall}{Precision +Recall}=\frac{2 TP}{2 TP + FP + FN}$ | (4) |
| --- | --- |

Precision describes the proportion of $TP$ to all positively predicted values as calculated with:

| $precision=\frac{TP}{TP+FP}$ | (5) |
| --- | --- |

Recall, also known as the true positive rate, describes the proportion of $TP$ to all actually positive values as calculated with:

| $Recall=\frac{TP}{TP+FN}$ | (6) |
| --- | --- |

The optimal F1 score is 1 and the worst is 0. The F1 score does not account for $TN$. Therefore, it is not symmetrical if the classes are swapped. This causes a limitation similar to that of accuracy where both metrics only show reliable results when applied to balanced data sets. [[49]](https://www.zotero.org/google-docs/?JGmHRm)

### **Mathews correlation coefficient (MCC)**

A metric that addresses the limitation of the previously mentioned metrics regarding imbalanced data is the Mathews correlation coefficient, which considers all elements of the so-called confusion matrix, $TP$, $TN$, $FP$, and $FN$. It is calculated as:

| $MCC=\frac{TP・TN-FP・FN}{\sqrt{(TP+FP)・(TP+FN)・(TN+FP)・(TN+FN)}}$ | (7) |
| --- | --- |

Its best score is 1 and its worst is -1, with 0 being the results of randomly guessing the classes. [[49,51]](https://www.zotero.org/google-docs/?OP5Uiy) While MCC is generally reliable, it relies on a threshold to distinguish positive and negative predictions, which may not be optimal in every application. Additionally, it can be undefined in cases where certain elements of the confusion matrix are zero, though mathematical adjustments can address this issue. MCC does not give insight into specific areas where a model fails; for instance, it cannot indicate whether issues arise from false positives or false negatives. However, when used alongside individual confusion matrix rates, MCC remains a strong and consistent metric for binary classification performance. [[49,51]](https://www.zotero.org/google-docs/?AZzWj2)

## **Regression metrics**

### **Mean absolute error (MAE)**

By comparing the true value $\hat{y}_{i}$ against the predicted value $y_{i}$ for all predictions that have a total number of n the MAE can be calculated with:

| $MAE=\frac{\sum_{i=1}^{n} \left\vert y_{i}-\hat{y}_{i} \right\vert}{n}$ | (8) |
| --- | --- |

### **Mean squared error (MSE)**

The MSE is calculated similar to the MAE but here the square of the error is used as in:

| $MSE=\frac{\sum_{i=1}^{n} \left( y_{i}-\hat{y}_{i} \right)^{2}}{n}$ | (9) |
| --- | --- |

### **Pearson correlation coefficient (PCC)**

The Pearson correlation coefficient allows to assess, how large the linear dependencies of two variables x and y is by calculating the ratio between the covariance of both variables with their standard deviations:

| $PCC=\frac{\sum_{i=1}^{n} \left( x_{i}-\overline{x} \right)\left( y_{i}-\overline{y} \right)}{\sqrt{\sum_{i=1}^{n} \left( x_{i}-\overline{x} \right)^{2}\left( y_{i}-\overline{y} \right)^{2}}}$ | (10) |
| --- | --- |

### **Median absolute percent error (MAPE)**

The MAPE is calculated similar to the MAE but rather than allowing the scale of the individual errors to impact the calculation they are normalized as percentage errors:

| $MAPE=\frac{\sum_{i=1}^{n} \left\vert\frac{y_{i}-\hat{y}_{i}}{y_{i}} \right\vert}{n}$ | (11) |
| --- | --- |

[1] Vermeulen, A. F., (2020). *Supervised learning: Using labeled data for insights*. Industrial Machine Learning: Using Artificial Intelligence as a Transformational Disruptor, 63-136 doi: 10.1007/978-1-4842-5316-8_4

[2] Celebi, M. E., & Aydin, K. (Eds.). (2016). *Unsupervised learning algorithms* (Vol. 9, p. 103). Cham: Springer, doi: 10.1007/978-3-319-24211-8

[3] Wiering, M. A., & Van Otterlo, M. (2012). Reinforcement learning. *Adaptation, learning, and optimization*, ***12***(3), 729, doi: 10.1007/978-3-642-27645-3

[4] Temple, P., & Perrouin, G. (2023, January). Explicit or Implicit? On Feature Engineering for ML-based Variability-intensive Systems. In *Proceedings of the 17th International Working Conference on Variability Modelling of Software-Intensive Systems*, 91-93, doi: 10.1145/3571788.3571804

[5] Rodríguez-Pérez, R., & Bajorath, J. (2019). *Interpretation of compound activity predictions from complex machine learning models using local approximations and shapley values*. Journal of medicinal chemistry, **63**(16), 8761-8777, doi: 10.1021/acs.jmedchem.9b01101

[6] Bisong, E. (2019). *What Is Deep Learning? Building Machine Learning and Deep Learning Models on Google Cloud Platform: A Comprehensive Guide for Beginners*, 327-329, doi: 10.1007/978-1-4842-4470-8_27

[7] Polson, N. G., & Sokolov, V. O. (2018). *Deep learning*. arXiv preprint arXiv:1807.07987, doi: 10.1002/9781118445112.stat08171

[8] Vogt, M. (2019). *An overview of deep learning and its applications*. Fahrerassistenzsysteme 2018: Von der Assistenz zum automatisierten Fahren 4. Internationale ATZ-Fachtagung Automatisiertes Fahren, 178-202, doi: 10.1007/978-3-658-23751-6_17

[9] Bhattacharyya, S., Snasel, V., Hassanien, A. E., Saha, S., & Tripathy, B. K. (Eds.). (2020). *Deep Learning: Research and Applications* (Vol. 7). Walter de Gruyter GmbH & Co KG, doi: 10.1515/9783110670905

[10] LeCun, Y., Bengio, Y., & Hinton, G. (2015). *Deep learning*. Nature, **521**(7553), 436-444, doi: 10.1038/nature14539

[11] Hochreiter, S. (1997). *Long Short-term Memory*. Neural Computation MIT-Press, doi: 10.1162/neco.1997.9.8.1735

[12] Guan, S., Moran, M. F., & Ma, B. (2019). *Prediction of lc-ms/ms properties of peptides from sequence by deep learnin*[s]*. Molecular & Cellular Proteomics, **18**(10), 2099-2107, doi: 10.1074/mcp.TIR119.001412

[13] Yi, H. C., You, Z. H., Zhou, X., Cheng, L., Li, X., Jiang, T. H., & Chen, Z. H. (2019). *ACP-DL: a deep learning long short-term memory model to predict anticancer peptides using high-efficiency feature representation*. Molecular Therapy-Nucleic Acids, **17**, 1-9, doi: 10.1016/j.omtn.2019.04.025

[14] Zeng, W. F., Zhou, X. X., Willems, S., Ammar, C., Wahle, M., Bludau, I., ... & Mann, M. (2022). *AlphaPeptDeep: a modular deep learning framework to predict peptide properties for proteomics*. Nature Communications, **13**(1), 7238, doi: 10.1038/s41467-022-34904-3

[15] Kuttichira, D. P., Verma, B., Rahman, A., & Wang, L. (2023). *Novel Automatic Deep Learning Feature Extractor with Target Class Specific Feature Explanations*. In 2023 International Joint Conference on Neural Networks (IJCNN). IEEE, 1-8, doi: 10.1109/IJCNN54540.2023.10191143

[16] Grasso, S., Dabene, V., Hendriks, M. M., Zwartjens, P., Pellaux, R., Held, M., ... & van Rij, T. (2023). *Signal peptide efficiency: from high-throughput data to prediction and explanation*. ACS synthetic biology, **12**(2), 390-404, doi: 10.1021/acssynbio.2c00328

[17] Zhou, Z., Yeung, W., Soleymani, S., Gravel, N., Salcedo, M., Li, S., & Kannan, N. (2024). *Using explainable machine learning to uncover the kinase–substrate interaction landscape*. Bioinformatics, **40**(2), btae033, doi: 10.1093/bioinformatics/btae033

[18] Yeung, D., Spicer, V., Zahedi, R. P., & Krokhin, O. (2023). *Exploring the variable space of shallow machine learning models for reversed-phase retention time prediction*. Computational and Structural Biotechnology Journal, **21**, 2446-2453, doi: 10.1016/j.csbj.2023.02.047

[19] Samek, W. (2023). *Explainable deep learning: concepts, methods, and new developments*. In Explainable Deep Learning AI, Academic Press, 7-33, doi: 10.1016/B978-0-32-396098-4.00008-9

[20] Santorsola, M., & Lescai, F. (2023). *The promise of explainable deep learning for omics data analysis: Adding new discovery tools to AI*. New Biotechnology, **77**, 1-11, doi: 10.1016/j.nbt.2023.06.002

[21] Chakraborty, C., Bhattacharya, M., Pal, S., & Lee, S. S. (2024). *From machine learning to deep learning: Advances of the recent data-driven paradigm shift in medicine and healthcare*. Current Research in Biotechnology, **7**, 100164, doi: 10.1016/j.crbiot.2023.100164

[22] Siemers, F. M., Feldmann, C., & Bajorath, J. (2022). *Minimal data requirements for accurate compound activity prediction using machine learning methods of different complexity*. Cell Reports Physical Science, **3**(11), doi: 10.1016/j.xcrp.2022.101113

[23] Bao, D., Shu, Q., Ning, B., Tang, M., Liu, Y., Wong, N., ... & Fan, J. (2024). *Improving Targeted Mass Spectrometry Data Analysis with Nested Active Machine Learning*. Advanced Intelligent Systems, **2300773**, doi: 10.1002/aisy.202300773

[24] Chung, H., Park, C., Kang, W. S., & Lee, J. (2021). *Gender bias in artificial intelligence: severity prediction at an early stage of COVID-19*. Frontiers in Physiology, **12**, 778720, doi: 10.3389/fphys.2021.778720

[25] Gharawi, A. A., Alsubhi, J., & Ramaswamy, L. (2022). *Impact of Labeling Noise on Machine Learning: A Cost-aware Empirical Study*. In 2022 21st IEEE International Conference on Machine Learning and Applications (ICMLA). IEEE, 936-939, doi: 10.1109/ICMLA55696.2022.00156

[26] Gupta, S., & Gupta, A. (2019). *Dealing with noise problem in machine learning data-sets: A systematic review*. Procedia Computer Science, **161**, 466-474, doi: 10.1016/j.procs.2019.11.146

[27] Al-Gethami, K. M., Al-Akhras, M. T., & Alawairdhi, M. (2021). *Empirical evaluation of noise influence on supervised machine learning algorithms using intrusion detection datasets*. Security and Communication Networks, **2021**(1), 8836057, doi: 10.1155/2021/8836057

[28] Ying, X. (2019). *An overview of overfitting and its solutions. In Journal of physics: Conference series.* IOP Publishing**, 1168**, 22022, doi: 10.1088/1742-6596/1168/2/022022

[29] Bernett, J., Blumenthal, D. B., Grimm, D. G., Haselbeck, F., Joeres, R., Kalinina, O. V., & List, M. (2024). *Guiding questions to avoid data leakage in biological machine learning applications*. Nature Methods, **21**(8), 1444-1453, doi: 10.1038/s41592-024-02362-y

[30] Black, A., & van Nederpelt, P. (2020). *Dimensions of data quality (DDQ)*. DAMA NL Foundation, 1-113, url: https://dama-nl.org/wp-content/uploads/2020/09/DDQ-Dimensions-of-Data-Quality-Research-Paper-version-1.2-d.d.-3-Sept-2020.pdf

[31] Wang, Q., Ma, Y., Zhao, K., & Tian, Y. (2020). *A comprehensive survey of loss functions in machine learning*. Annals of Data Science, 1-26, doi: 10.1007/s40745-020-00253-5

[32] Cordeiro, F. R., & Carneiro, G. (2020). *A survey on deep learning with noisy labels: How to train your model when you cannot trust on the annotations?*. In 2020 33rd SIBGRAPI conference on graphics, patterns and images (SIBGRAPI) (pp. 9-16). IEEE, doi: 10.1109/SIBGRAPI51738.2020.00010

[33] Zhang, Z., & Sabuncu, M. (2018). *Generalized cross entropy loss for training deep neural networks with noisy labels*. Advances in neural information processing systems, **31**.

[34] Ma, X., Huang, H., Wang, Y., Romano, S., Erfani, S., & Bailey, J. (2020). *Normalized loss functions for deep learning with noisy labels*. In International conference on machine learning (pp. 6543-6553). PMLR.

[35] Zhou, X., Liu, X., Zhai, D., Jiang, J., & Ji, X. (2023). *Asymmetric loss functions for noise-tolerant learning: Theory and applications*. IEEE Transactions on Pattern Analysis and Machine Intelligence, **45**(7), 8094-8109, doi: 10.1109/TPAMI.2023.3236459

[36] Gonzalez, S., & Miikkulainen, R. (2020). *Improved training speed, accuracy, and data utilization through loss function optimization*. In 2020 IEEE congress on evolutionary computation (CEC) (pp. 1-8). IEEE, doi: 10.1109/CEC48606.2020.9185777

[37] Wilburn, D.B., Shannon, A.E., Spicer, V., Richards, A.L., Yeung, D., Swaney, D.L., Krokhin, O.V. and Searle, B.C. (2023). *Deep learning from harmonized peptide libraries enables retention time prediction of diverse post translational modifications*. BioRxiv, 2023-05, doi: 10.1101/2023.05.30.542978

[38] Jumper, J., Evans, R., Pritzel, A., Green, T., Figurnov, M., Ronneberger, O., ... & Hassabis, D. (2021). *Highly accurate protein structure prediction with AlphaFold*. Nature, **596**(7873), 583-589, doi: 10.1038/s41586-021-03819-2

[39] Abramson, J., Adler, J., Dunger, J., Evans, R., Green, T., Pritzel, A., ... & Jumper, J. M. (2024). *Accurate structure prediction of biomolecular interactions with AlphaFold 3*. Nature, 1-3, doi: 10.1038/s41586-024-07487-w

[40] Amari, S. I. (1993). *Backpropagation and stochastic gradient descent method*. Neurocomputing, **5**(4-5), 185-196, doi: 10.1016/0925-2312(93)90006-O

[41] 3Blue1Brown, *Neural Networks*, YouTube, 2017. url: https://www.youtube.com/watch?v=aircAruvnKk&list=PLZHQObOWTQDNU6R1_67000Dx_ZCJB-3pi

[42] Wang, J., & Chen, Y. (2023). *Introduction to transfer learning: algorithms and practice*. Springer Nature, doi: 10.1007/978-981-19-7584-4

[43] Pan, S. J., & Yang, Q. (2009). *A survey on transfer learning*. IEEE Transactions on knowledge and data engineering, **22**(10), 1345-1359, doi: 10.1109/TKDE.2009.191

[44] Wang, Y., Yao, Q., Kwok, J. T., & Ni, L. M. (2020). *Generalizing from a few examples: A survey on few-shot learning*. ACM computing surveys (csur), **53**(3), 1-34, doi: 10.1145/3386252

[45] Wang, W., Zheng, V. W., Yu, H., & Miao, C. (2019). *A survey of zero-shot learning: Settings, methods, and applications*. ACM Transactions on Intelligent Systems and Technology (TIST), **10**(2), 1-37, doi: 10.1145/3293318

[46] Kunanbayev, K., Temirbek, I., & Zollanvari, A. (2021). *Complex encoding*. In 2021 International Joint Conference on Neural Networks (IJCNN) (pp. 1-6). IEEE, doi: 10.1109/IJCNN52387.2021.9534094

[47] Neelima, A., & Mehrotra, S. (2023). *A comprehensive review on word embedding techniques*. In 2023 International Conference on Intelligent Systems for Communication, IoT and Security (ICISCoIS) (pp. 538-543). IEEE, doi: 10.1109/ICISCoIS56541.2023.10100347

[48] Webster, J. J., & Kit, C. (1992). *Tokenization as the initial phase in NLP*. In COLING 1992 volume 4: The 14th international conference on computational linguistics, doi: 10.3115/992424.992434

[49] Chicco, D., & Jurman, G. (2020). *The advantages of the Matthews correlation coefficient (MCC) over F1 score and accuracy in binary classification evaluation*. BMC genomics, **21**, 1-13, doi: 10.1186/s12864-019-6413-7

[50] Uddin, M. F. (2019). *Addressing accuracy paradox using enhanched weighted performance metric in machine learning*. In 2019 Sixth HCT Information Technology Trends (ITT) (pp. 319-324). IEEE, doi: 10.1109/ITT48889.2019.9075071

[51] Chicco, D., & Jurman, G. (2023). *The Matthews correlation coefficient (MCC) should replace the ROC AUC as the standard metric for assessing binary classification*. BioData Mining, **16**(1), 4, doi: 10.1186/s13040-023-00322-4

[52] Lobo, J. M., Jiménez‐Valverde, A., & Real, R. (2008). *AUC: a misleading measure of the performance of predictive distribution models*. Global ecology and Biogeography, **17**(2), 145-151, doi: 10.1111/j.1466-8238.2007.00358.x
